# A new perspective of molecular diffusion by nuclear magnetic resonance

**DOI:** 10.1038/s41598-023-27389-7

**Published:** 2023-01-30

**Authors:** Giulio Costantini, Silvia Capuani, Francis Allen Farrelly, Alessandro Taloni

**Affiliations:** 1 Istituto Sistemi Complessi, Consiglio Nazionale delle Ricerche, UOS Sapienza, 00185 Rome, Italy; 2 Istituto Sistemi Complessi, Consiglio Nazionale delle Ricerche, via dei Taurini 19, 00185 Rome, Italy

**Keywords:** Applied physics, Fluid dynamics, Solution-state NMR

## Abstract

The diffusion-weighted NMR signal acquired using Pulse Field Gradient (PFG) techniques, allows for extrapolating microstructural information from porous materials and biological tissues. In recent years there has been a multiplication of diffusion models expressed by parametric functions to fit the experimental data. However, clear-cut criteria for the model selection are lacking. In this paper, we develop a theoretical framework for the interpretation of NMR attenuation signals in the case of Gaussian systems with stationary increments. The full expression of the Stejskal–Tanner formula for normal diffusing systems is devised, together with its extension to the domain of anomalous diffusion. The range of applicability of the relevant parametric functions to fit the PFG data can be fully determined by means of appropriate checks to ascertain the correctness of the fit. Furthermore, the exact expression for diffusion weighted NMR signals pertaining to Brownian yet non-Gaussian processes is also derived, accompanied by the proper check to establish its contextual relevance. The analysis provided is particularly useful in the context of medical MRI and clinical practise where the hardware limitations do not allow the use of narrow pulse gradients.

## Introduction

Introduced by Stejskal and Tanner in 1965^1^, the molecular diffusion measurement by nuclear magnetic resonance (NMR), in parallel to the development of sophisticated experiments in soft condensed matter^2,3^ and porous materials^4^, entered into clinical practice at the beginning of the second millennium, resulting in a revolution in clinical radiology^5^ and neuroimaging investigations^6^.

The hodological approach to molecular diffusion makes NMR the only technique capable of inferring microscopic structures in a non-invasive, non-destructive, and radiation-free modality. However, NMR measurements capture a statistical average of microscopic dynamical features over a large ensemble of molecules, rather than their individual pathways. This inherent probabilistic description must necessarily rely on assumptions regarding the dynamics of molecular diffusion. In turn, these assumptions are implemented in mathematical models yielding the parametric functions that are used to fit the diffusion-weighted (DW) NMR signals. The NMR acquisition sequences for obtaining a DW signal are based on applying pulse field gradient (PFG)^1^ along different directions $\vec {q}$^2,4,6^, where $|\vec {q}|=\gamma g\delta /(2\pi )$, $\gamma$ is the nuclear gyromagnetic ratio, *g* is the magnetic field gradient strength and $\delta$ is the duration of the applied pulse. Due to molecular diffusion, this signal $S(\vec {q})$ is attenuated compared to the signal *S*(0) acquired without gradient application. In general, the functional form of the $S(\vec {q})/S(0)$ DW-NMR signal decay depends on the interplay between the type of molecular diffusion and the media where the diffusion occurs.

Conventional diffusion NMR, such as diffusion weighted imaging (DWI)^7^ and Diffusion Tensor Imaging (DTI)^8^, largely used in current clinical applications, is based on the simplifying assumption that water molecules perform normal (Brownian) self-diffusion inside the tissues, i.e. their dynamics are characterized by a molecular mean squared displacement (MSD) which grows linearly in time^9,10^. A further fundamental assumption that is usually accepted in classical NMR signal representation theory is that the motion propagator (MP) is Gaussian. The genesis of these two assumptions dates back to the Torrey description of self-diffusion as a “transport of magnetization”^11^ in the form introduced by Abragam^12^, obtained by the insertion of an additional term into the Bloch equation. However, with the improvement of the experimental acquisition set-ups, it appears clear that the diffusion in most human tissues, soft matter, and porous systems, is far from being normal and/or Gaussian, exhibiting more complex and diversified scenarios, due to the concatenation of the diffusion dynamics and to the contribution of structural inhomogeneities, such as component mixtures and/or confining regions. Therefore, several approaches have been developed in the attempt to find parametric functions that best fit the experimental data and provide new parameters characterizing the tissues microstructures. The goal is to increase sensitivity and resolution of diagnostics NMR, obtaining complementary information compared to conventional DWI and DTI metrics^6^. Moreover, a productive strategy to be able to extract relevant information from diffusion is, certainly, a multidirectional approach using different techniques. In this regard, however, many works^13–16^ use models that assume a Gaussian MP and Brownian diffusion.

The disengagement from the Gaussian assumption hinges upon the context of the cumulant expansion of the DW-NMR attenuation signals^9^. If the cumulant expansion is truncated to the second order, the conventional picture applies, resulting in the so-called “Gaussian approximation in cumulant expansion”^17–20^. Conversely, any deviation from the Gaussian behavior can be quantified using a convenient dimensionless expression of the MP fourth cumulant called the excess kurtosis, often shortened to “kurtosis”^21–23^. This approach is particularly promising in clinical investigations^24^. Moreover, the deviation from the Gaussianity can be readily identified in the narrow-pulse gradient (NPG) experiments, i.e. when $\delta \ll \Delta$. In this case the normalized NMR signal turns out to be the Fourier transform in $|\vec {q}|$ of the MP^2,25^.

Anomalous diffusion is a generalization of the Brownian diffusion, in the sense that the molecular MSD scales as $t^{\alpha }$ with $0<\alpha <2$ and $\alpha \ne 1$^26^. In the past 30 years, a large number of experimental results have been accumulated, providing evidence that NMR attenuation may exhibit systematic deviations from the pure (Debye) exponential decay, arising from the assumption of normal molecular self-diffusion^27–33^. At the end of the last century it was observed that a stretched-exponential or Kohlrausch–Williams–Watts (KWW) parametric function could better describe the decay of $S(\vec {q})/S(0)$^34^, a feature that was later attributed to the anomalous diffusion dynamics of water molecules inside complex self-similar structures^34,35^. Hence, in heterogeneous systems the Brownian assumption was replaced by other assumptions, searching for more effective models to reproduce the anomalous microscopic transport dynamics. In all likelihood, the continuous time random walk (CTRW)^36^ is the most popular among the anomalous diffusion models^37–41^ used in NMR, due to its connection both to Lévy statistics^34^ and to the fractional diffusion equation, regarded as the natural generalization of the classical Bloch–Torrey equation^42–45^. It is worth noticing that the CTRW also surmounts the Gaussianity assumption, as the MP, in this case, is given by a Fox function^46^. Although less fashionable than CTRW, different microscopical models have been postulated to justify the anomalous diffusion appearing in the KWW decay: diffusion on fractal structures^35,47^, fractional motion models^48^, anomalous diffusion with Gaussian MP^49–52^ or processes fulfilling the generalized fluctuation–dissipation theorem such as those generated by a fractional Langevin equation^53^. However, the methodologies used, the results and their interpretation have often aroused doubts, even questioning the fact that biological water in tissues can effectively diffuse anomalously^54–56^.

The progressive departure from the hypothesis subtending the unbounded free diffusion is evident in the case of NMR signals arising from compartmentalized system, where water diffusion takes place in restricted geometries. In these cases, the exact solution of the Bloch–Torrey equation and the ensuing attenuation signal form gets complicated expressions^57,58^. Indeed, the first attempts to solve the Bloch–Torrey equation were directed toward perturbative approaches^59,60^, highlighting the presence of two distinct dynamical regimes in the spin-echo signal decay. If time is short compared to the time required from diffusion from one boundary to the other, the resulting equation and the corresponding outcome reduce to those representing unrestricted diffusion. For longer times instead, the bulk diffusion coefficient *D* is reduced to an apparent diffusion coefficient, $D_{eff}$, accounting for the presence of the microstructure. $D_{eff}$, however, reveals a non-trivial dependence on the time which undoubtedly unveils the violation of the hypothesis of Brownian diffusion. On the other side, the Gaussian character of the propagator is still preserved, as the $g^2$ dependence of the DW signal proves. This is ultimately clarified by the fact that the identical limiting expressions for the spin-echo amplitude were derived using a completely different technique, namely the Gaussian phase approximation (which coincides with the Gaussian approximation in cumulant expansion )^61,62^. In 1991 the exact solution of the Bloch-Torrey equation was provided by Stoller et al., although limited to one dimensional bounded domains^63^. In this thorough study it was implicit that another regime could arise, when the intrinsic length scale of dynamics depending on the field gradient ($\propto g^{-1/3}$) is much smaller than the average size characterizing the microstructure. This important point was extensively developed in^64^ where it was demonstrated that in this regime the Gaussian phase approximation does not hold and higher cumulants become important. This scenario, named localization regime, has attracted more and more interest in recent years, since it has become clear that it constitute an universal feature of the Bloch–Torrey equation (see Ref.^57^ and references therein).

This manuscript aims at providing a useful toolkit for an NMR-scientist who is facing the twofold problem of using the correct fitting formula for the PFG NMR attenuation $S(\vec {q})/S(0)$ and, at the same time, inferring the underlying details of the molecular dynamics. We show how to implement a sequence of simple checks leading to the identification of the correct analytical expression for the analysis of PFG DW-NMR signal, in various diffusion contexts. We provide a “recipe” that can help to understand the type of dynamics “before” applying the diffusion models, or the (dynamical) domain of applicability of a certain microscopical model. For the sake of clarity, different parametric formulas are derived from different molecular diffusion models: one for the Brownian Gaussian processes with stationary increments^10^, one for the anomalous Gaussian processes with stationary increments^65^, and one for Brownian yet non-Gaussian diffusion-like systems^66^. A set of easy-to-implement checks, hereby called validation rules, define the range of applicability of each one of these formulas. In addition we show how existing and apparently scattered results in literature fit neatly into our framework.

The paper is organized as follows. In “Gaussian approximation in cumulant expansion” we recall the Gaussian approximation in cumulant expansion, where the assumptions of Gaussianity and stationarity of increments of the stochastic processes are clearly formulated. Moreover, by applying the PFG sequence, we furnish a clear-cut benchmark to determine if the self-diffusion is normal or anomalous. In “Normal diffusion” and “Anomalous diffusion” we discuss separately the two cases, providing the correct formulas to fit the DW-NMR attenuation signals. In “Checking the assumptions: model’s discriminative power” we question the applicability of these formulas to systems where they are not compelling, i.e., which do not fulfill the Gaussianity and the stationarity of increments assumptions. Furthermore, we furnish the precise criteria to discern Gaussian stationary processes from any other. In particular in “PFG NMR signal for the superstatistical model for Brownian yet non-Gaussian diffusion” we furnish the correct fitting expression for system displaying the Brownian yet non-Gaussian diffusion. In “Validation rules: a practical example” a practical example is reported, considering the PFG signal of free water diffusion and water diffusion in packed microspheres of different diameters. Our conclusions are summarized in “Conclusions”.

## Gaussian approximation in cumulant expansion

The transverse magnetization of a spin-bearing particle (or molecule) can be expressed via the unit complex vector $e^{-i\phi }$, where the phase built up during the motion in a magnetic field gradient is given by^67^

1 $$\begin{aligned} \phi (t)=\gamma \int _0^t dt' {\textbf{r}}(t')\cdot {\textbf{G}}(t'). \end{aligned}$$

Here $\gamma$ is the gyromagnetic ratio, ${\textbf{r}}(t)$ is the particle/molecule position and ${\textbf{G}}(t)$ is the uniform magnetic field gradient. It is clear that the fluctuation of $\phi$ results from the stochastic change of the particle location, which we imagine continuous and differentiable, obeying the following equation

2 $$\begin{aligned} {\textbf{r}}(t)={\textbf{r}}(0)+\int _0^tdt' {\textbf{v}}(t'), \end{aligned}$$

with ${\textbf{v}}(t)$ the spin velocity and ${\textbf{r}}(0)$ the initial spin position measured from the gradient center. After integration by parts, Eq. (1) becomes^17–19,21,22^, at the time of spin refocusing $t=TE$ (*time echo*),

3 $$\begin{aligned} \phi (TE)= -\gamma \int _0^{TE} dt' {\textbf{v}}(t')\cdot {\textbf{F}}(t'), \end{aligned}$$

where ${\textbf{F}}(t)=\int _0^t dt' {\textbf{G}}(t')$ and ${\textbf{F}}(TE)=0$. The NMR signal attenuation is defined as the ensemble average spin echo amplitude, properly normalized:

4 $$\begin{aligned} \frac{S(TE)}{S(0)}=\langle e^{i\gamma \int _0^{TE} dt' {\textbf{v}}(t')\cdot {\textbf{F}}(t')}\rangle , \end{aligned}$$

where *S*(0) is the initial value of the signal. Taking the logarithm of this expression and performing its cumulant expansion^9,17–22,53^, we obtain

5 $$\begin{aligned} \ln \frac{S(TE)}{S(0)} \simeq -\frac{\gamma ^2}{2}\mathop {\int }\limits _0^{TE} u_2(t_1,t_2)F_2(t_1,t_2)dt_1dt_2 +\frac{\gamma ^4}{4!}\mathop {\int }\limits _0^{TE} u_4(t_1,t_2,t_3,t_4)F_4(t_1,t_2,t_3,t_4)\,dt_1dt_2dt_3dt_4+\cdots , \end{aligned}$$

where $F_2(t_1,t_2)\equiv F(t_1)F(t_2)$ and $F_4(t_1,t_2,t_3,t_4)\equiv F(t_1)F(t_2)F(t_3)F(t_4)$. $u_2()$ and $u_4()$ represent respectively the second and fourth cumulants of the molecular velocity, in the simpler situation where the gradient is in the *x*-direction only (the extension to the three dimensional tensorial structure is a delicate aspect that can be treated to measure the correlations between the differing components of the displacements^2,21^). We neglected both higher-order cumulants, since we imagine the field amplitude small enough, and the odd-order terms, as they do not contribute to the signal attenuation^22^.

Finally, we introduce the fundamental assumptions characterizing the one-dimensional stochastic process *v*(*t*). We consider a zero-mean molecular velocity whose correlation function is stationary (stationarity of increments), i.e.

6 $$\begin{aligned} u_2(t_1,t_2)\equiv \langle v(t_1)v(t_2)\rangle =C\left( |t_1-t_2|\right) , \end{aligned}$$

and whose fourth cumulant is negligible:

7 $$\begin{aligned} u_4(t_1,t_2,t_3,t_4)\simeq 0. \end{aligned}$$

Thanks to Eq. (7) and to the zero flow condition, the DW-NMR signal assumes the following form^17–21,53,68,69^

8 $$\begin{aligned} \ln \frac{S(TE)}{S(0)} \simeq -\frac{\gamma ^2}{2}\mathop {\int }\limits _0^{TE}dt_1 \mathop {\int }\limits _0^{TE}dt_2 \langle v(t_1)v(t_2)\rangle F(t_1)F(t_2), \end{aligned}$$

which becomes

9 $$\begin{aligned} \ln \frac{S(TE)}{S(0)} \simeq -\gamma ^2\mathop {\int }\limits _0^{TE}C(s) ds\mathop {\int }\limits _s^{TE} F(t')F(t'-s)\,dt' \end{aligned}$$

after implementing the stationarity hypothesis (6).

The second order cumulant expansion can be performed also by adopting the phase definition (1)^2,49–51,70–73^

10 $$\begin{aligned} \ln \frac{S(TE)}{S(0)} \simeq -\frac{\gamma ^2}{2}\mathop {\int }\limits _0^{TE}dt_1 \mathop {\int }\limits _0^{TE}dt_2 \langle x(t_1)x(t_2)\rangle G(t_1)G(t_2), \end{aligned}$$

where $\langle x(t)\rangle =0$ (stagnant liquids). It is clear that Eq. (10) is equivalent to Eq. (8) and, once one assumes the stationarity of the position autocorrelation function, the analogous of Eq. (9) reads

11 $$\begin{aligned} \ln \frac{S(TE)}{S(0)} \simeq -\gamma ^2\mathop {\int }\limits _0^{TE} \langle x(0)x(s)\rangle ds \mathop {\int }\limits _s^{TE} G(t')G(t'-s)\,dt'. \end{aligned}$$

### Normal and anomalous DW-NMR signal in a Pulsed field gradient (PFG) experiment

**Figure 1:**
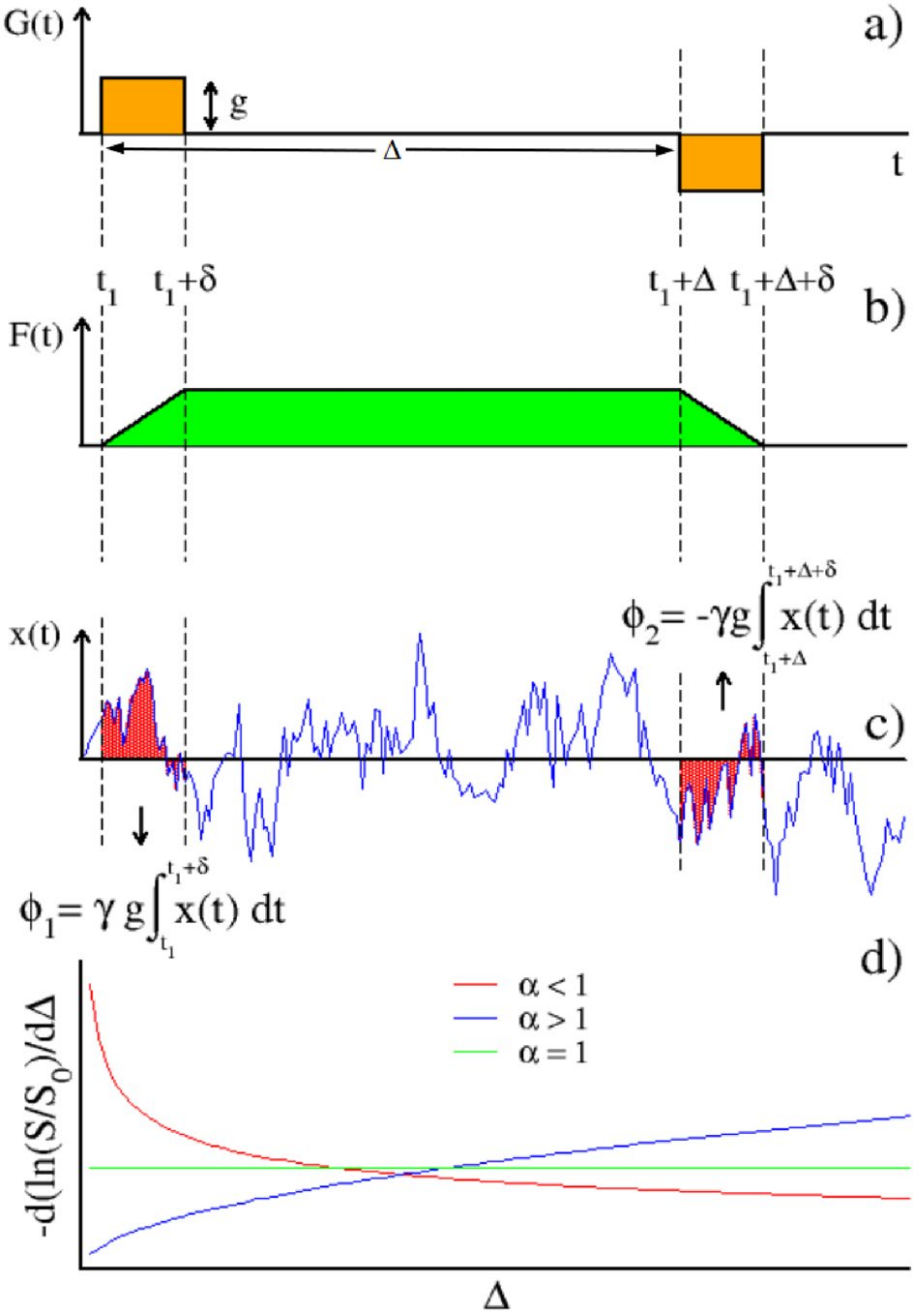
PFG and anomalous diffusion. Panel (**a**) A schematic representation of the couple of magnetic field gradients in PFG-based sequence. Panel (**b**) Integral function *F*(*t*) of the gradient in panel (**a**). Panel (**c**) phase accumulating during the gradient pulses: the same scheme has been used to obtain the DW-NMR signal from stochastic trajectories (see “Methods” section). Panel (**d**) Expected behavior of the derivative of the logarithm of the normalized echo amplitude for different values of the anomalous diffusion exponent $\alpha$. For subdiffusive processes the derivative is a decaying function of $\Delta$, for superdiffusive processes it is an increasing function, whilst for normal diffusing systems it is constant.

In the PFG-based experiment^1^ after a radiofrequency $\pi /2$ pulse which brings the magnetization in the transverse plane to the direction of the static magnetic field $\vec {B}$, a short gradient pulse of amplitude *g* and duration $\delta$ confers phase shifts to the spins. A second equivalent pulse, after an intermediate $180^\circ$ radio-frequency pulse reverses the phase shifts to yield an unattenuated signal in the absence of any motions along the gradient. However, the molecular spins collisions during the diffusion time $\Delta$ between the two gradient pulses can cause unequal phase shifts, resulting in an attenuated NMR signal. Thus, once the *g* and $\delta$ values have been set, the NMR attenuation signal is a function of the magnetic-field gradient pulse interspacing $\Delta$. In PFG experiments, the same effect is achieved if the two pulses have an opposite sign, but without the $180^\circ$ radio-frequency pulse. Without loss of generality, in our treatment we will adopt the latter PFG pulses sequence (see Fig. 1a,b and Supplementary Fig.S1 in the supplementary online materials (SOM)). Furthermore, in the following we will not consider the effects of nuclear relaxation, as we will deal with the temporal duration of the PFG sequence which is much less than the relaxation time $T_2$^2^.

The explicit calculation of the Eq. (9) in case of PFG yields (see “Methods” section for details and SOM)

12 $$\begin{aligned} \begin{aligned} \ln \frac{S(\Delta )}{S(0)}&= -\gamma ^2g^2 \Biggl \{ \delta ^2\biggl [\int _0^\delta ds\, C(s) \left( \Delta -\frac{\delta }{3}\right) + \int _\delta ^\Delta ds\, C(s) \left( \Delta -s\right) \biggr ] - \int _0^\delta ds\, C(s) s^2\left( \delta -\frac{s}{3}\right) \\&\quad - \frac{1}{6}\int _{\Delta -\delta }^\Delta ds\, C(s) \left( \Delta -\delta -s\right) ^3 +\frac{1}{6}\int _{\Delta }^{\Delta +\delta } ds\, C(s) \left( \Delta +\delta -s\right) ^3 \Biggr \}. \end{aligned} \end{aligned}$$

Likewise (see the SOM), it is possible to derive the echo amplitude from Eq. (11):

13 $$\begin{aligned} \ln \frac{S(\Delta )}{S(0)}= & {} -\gamma ^2g^2\Biggl \{2\int _0^\delta ds\, \langle x(0)x(s)\rangle \left( \delta -s\right) \nonumber \\{} & {} + \int _{\Delta -\delta }^\Delta ds\, \langle x(0)x(s)\rangle \left( \Delta -\delta -s\right) - \int _{\Delta }^{\Delta +\delta } ds\, \langle x(0)x(s)\rangle \left( \Delta +\delta -s\right) \Biggr \}. \end{aligned}$$

Taking the derivative of both members of Eq. (12) we obtain

14 $$\begin{aligned} \frac{d}{d\Delta } \left( \ln \frac{S(\Delta )}{S(0)}\right)= & {} -\gamma ^2g^2\delta ^2\int _0^\Delta ds\, C(s)\nonumber \\{} & {} +\frac{\gamma ^2g^2}{2}\Biggl [-\int _{\Delta -\delta }^{\Delta }ds\,C(s)\left( \Delta -\delta -s\right) ^2+\int _{\Delta }^{\Delta +\delta }ds\,C(s)\left( \Delta +\delta -s\right) ^2\Biggr ]. \end{aligned}$$

Now, recalling that the relation between the mean square displacement and the velocity autocorrelation function of the process (2) is given by

15 $$\begin{aligned} \langle [x(t)-x(0)]^2\rangle =2\int _0^t ds\,C(s)(t-s), \end{aligned}$$

the following equality is valid in the limit of large gradient-field interspacing $\Delta$ ($\gtrsim 2\delta$):

16 $$\begin{aligned} \frac{d}{d\Delta } \left( \ln \frac{S(\Delta )}{S(0)}\right) \simeq -\frac{\gamma ^2g^2\delta ^2}{2}\frac{d}{d\Delta } \left( \langle [x\left( \Delta \right) -x(0)]^2\rangle \right) . \end{aligned}$$

The Eq. (16) is the linchpin of the first part of our analysis, because it allows to disclose the nature of the asymptotic diffusive dynamics, be normal or anomalous, by just looking at the derivative of the logarithm of the DW-NMR signal attenuation for a Gaussian process with stationary increments. Let us illustrate this point with the help of the theorem in^74^ (see also the discussion in Ref.^75^).

Suppose that the spins undergo normal diffusion, i.e.

17 $$\begin{aligned} \langle [x\left( \Delta \right) -x(0)]^2\rangle =2D\Delta . \end{aligned}$$

- Hence, $\int _0^\Delta ds\, C(s) \rightarrow D$ for $\Delta$ larger than the typical velocity correlation time. The derivative of the logarithm of the DW-NMR signal would then attain a constant value (Fig.1d).
- Now, let us imagine that the spins behave anomalously:
  18 $$\begin{aligned} \langle [x\left( \Delta \right) -x(0)]^2\rangle =2D_\alpha \Delta ^\alpha , \end{aligned}$$
  with $0<\alpha <2$. Therefore $\int _0^\Delta ds\, C(s) \rightarrow \alpha D_\alpha \Delta ^{\alpha -1}$ for $\Delta \rightarrow \infty$, ergo
  19 $$\begin{aligned} C(s)\sim \alpha (\alpha -1)D_\alpha s^{\alpha -2}. \end{aligned}$$
- Subdiffusive processes characterized by $0<\alpha <1$ and antipersistency in the velocity correlation, would display $\int _0^\Delta ds\, C(s) \rightarrow 0$ for large $\Delta$ (Fig.1d).
- Superdiffusive processes with $1<\alpha <2$ and a positive velocity autocorrelation function, would be identified by $\int _0^\Delta ds\, C(s) \rightarrow \infty$ as $\Delta \rightarrow \infty$ (Fig.1d).

Thus, the quantity $-\frac{d}{d\Delta } \left( \ln \frac{S(\Delta )}{S(0)}\right)$ helps to assess the diffusive regime that in average a molecular system exhibits in time, if the velocity stochastic process fulfills the hypothesis (6)–(7). Furthermore, plugging the normal or anomalous form of *C*(*s*) into the expression (12) will furnish the analytical function for the correct interpretation of the signal attenuation.

## Normal diffusion

The determination of the molecular diffusion by means of the relations (8) or (10) has a long history^2,20,53,73^. Indeed, the classical Stejskal–Tanner spin-echo attenuation was recovered in the limit of large $\Delta$, if compared to the correlation time of migrations^2,20^.

In our framework, the PFG expression (12) for normal diffusing molecules can be calculated exactly.

### Brownian motion

The Brownian motion (BM) is the only process undergoing normal diffusion under the hypothesis (6) and (7). The underneath stochastic process connected to the Eq. (2) is given by the Langevin equation

20 $$\begin{aligned} {\dot{v}}(t)+\zeta v(t)= \xi _{BM}(t). \end{aligned}$$

The molecule mass here has been set equal to 1, while $\zeta$ is the viscous drag and the Gaussian white noise satisfies the following properties: $\langle \xi _{BM}(t)\rangle =0$ and $\langle \xi _{BM}(t_1)\xi _{BM}(t_2)\rangle =2k_BT\zeta \delta (t_1-t_2)$, where $k_B$ is the Boltzmann constant and $\delta (t)$ is the Dirac’s delta function. The spin velocity autocorrelation function can be easily calculated^2,20,53^:

21 $$\begin{aligned} C(s)=k_BT e^{-\zeta s}. \end{aligned}$$

**Figure 2:**
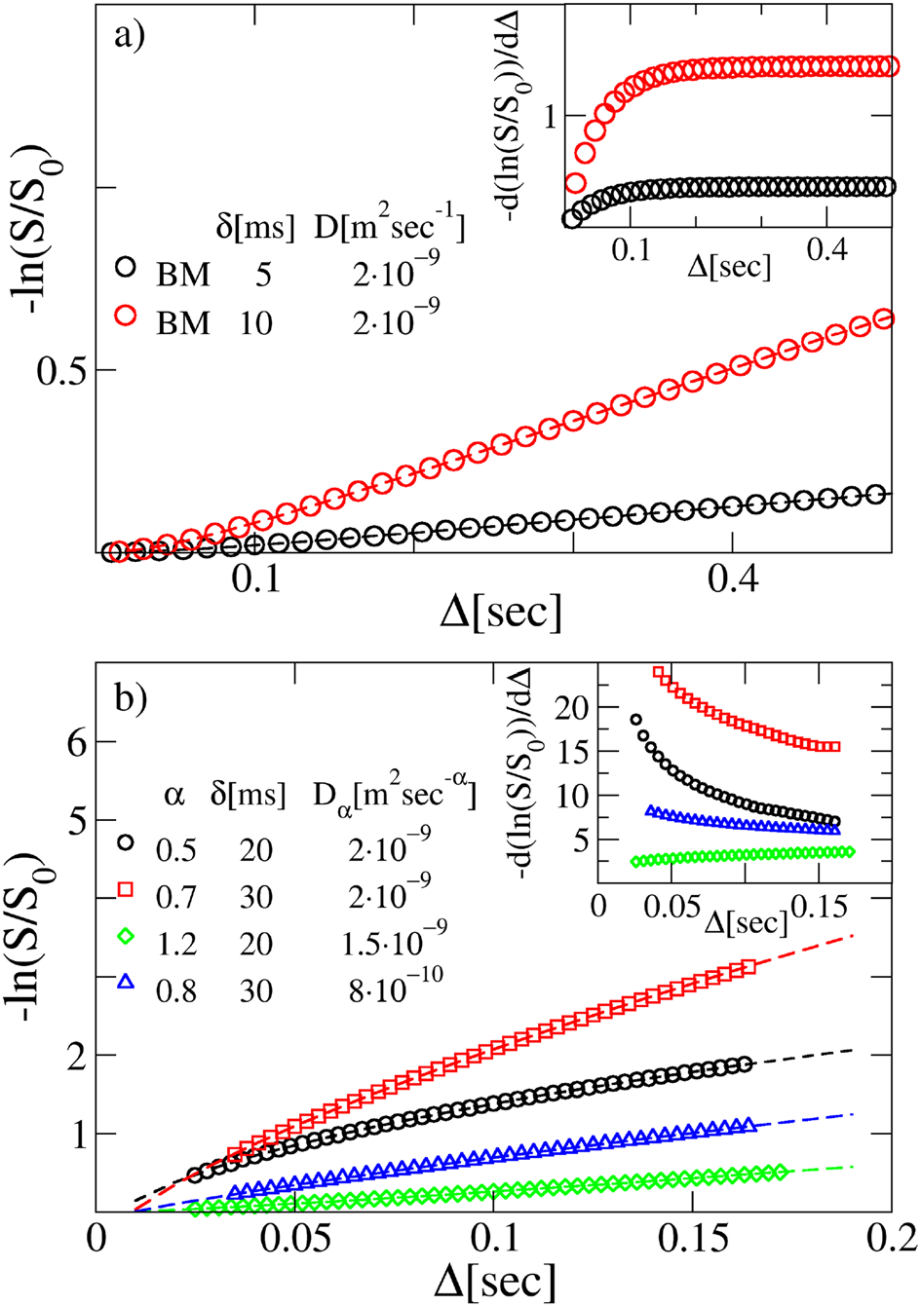
Panel (**a**) Normal diffusion. Main panel: comparison between synthetic DW-NMR signals obtained from normal diffusing trajectories and fitting curves obtained from Eq. (22) (dashed curves). The parameters of BM are $\zeta =20~s^{-1}$ and $g=0.01~T/m$. The estimated *D* and $\zeta$ are reported in Table 1. The fitted parameters are in excellent agreement with the expected values (see SOM). Inset: the constant trend of the derivative of the normalized DW-NMR signals is shown. Panel (**b**) Anomalous diffusion echo amplitudes. Main panel: comparison between different synthetic DW-NMR signals obtained from FBM subdiffusive and superdiffusive trajectories (symbols), and relative fitting curves obtained through Eq. (24) (dashed curves). The signals are obtained with a magnetic field gradient $g=0.01$ T/m. Inset : trend of the derivative of the logarithm of the normalized DW-NMR signals shown in the main plot. Superdiffusive (increasing trend as a function of the diffusion time) and subdiffusive (decreasing trend as a function of the diffusion time) systems are easily appraised.

By insertion of (21) into the signal attenuation (12), we obtain (see “Methods” section and SOM)

22 $$\begin{aligned} \ln \frac{S(\Delta )}{S(0)} = -\gamma ^2g^2\delta ^2D\left( \Delta -\frac{\delta }{3} \right) +2\gamma ^2g^2D\left\{ \frac{\delta }{\zeta ^2}-\frac{1-e^{-\delta \zeta } + e^{-\Delta \zeta } \left[ \cosh (\delta \zeta )-1\right] }{\zeta ^3}\right\} , \end{aligned}$$

where the diffusion coefficient is given by $D=\frac{k_BT}{\zeta }$. This result extends and amends the conclusions presented in previous references^2,20,73^, providing the generalization of the Stejskal–Tanner formula in terms of the generic gradient-pulse duration $\delta$, of the diffusing time $\Delta$ and of the velocity correlation time $\zeta ^{-1}$. Using the Eq. (22) for fitting DW-NMR attenuations coming from simulated Brownian trajectories, yields the correct estimates of the parameters *D* and $\zeta$ (see Fig. 2a and Table 1).

Thus the Eq. (22) is the correct expression for fitting DW-NMR attenuations coming from systems for which the Brownian motion is the significant model of diffusion. The two fitting parameters are *D* and $\zeta$, the latter of which allows to assess if the pulse gradient amplitude $\delta$ is short enough to assume that the velocity autocorrelation function can be approximated by a delta function ($\delta \zeta \gg 1$), or it has an exponential form such as that in Eq. (21).

**Table 1:** Fit parameters of the synthetic curves obtained from BM trajectories.

$\delta$ (s)	*g* (T/m)	Exact *D* (m$^2/$s)	Fitted *D* (m$^2/$s)	Exact $\zeta$ (s$^{-1}$)	Fitted $\zeta$ (s$^{-1}$)	*D* error $(\%)$	$\zeta$ error $(\%)$
0.005	0.01	$2\times 10^{-9}$	$2.010227 \times 10^{-9} \pm 1.40\times 10^{-13}$	20.0	$20.30564\pm 0.01309$	$0.51\%$	$1.53\%$
0.010	0.01	$2\times 10^{-9}$	$2.013836\times 10^{-9} \pm 0.43\times 10^{-13}$	20.0	$19.91906 \pm 0.00383$	$0.69\%$	$0.40\%$
0.010	0.02	$2\times 10^{-9}$	$2.033644\times 10^{-9} \pm 8.38\times 10^{-13}$	20.0	$19.07904 \pm 0.06802$	$1.68\%$	$4.60\%$

## Anomalous diffusion

The Gaussian approximation (8) was used in several NMR measurements of anomalous diffusion described in Eq. (18)^17,52^. Gradient modulation sequences different than PFG were hypothesized to determine the low-frequency information concerning the spectrum of the molecular velocity autocorrelation, which in the time domain exhibited long-time tails^2,17,68,69,76,77^. Moreover, since power-law decay such that in (19) also characterizes the molecular motion in compartments of limited size, the same modulated-gradient spin-echo method (MGSE) has been suggested for the interpretation of diffusion in restricted geometries^17–19,75,78^.

Our theory assesses that the PFG expression (12) is an important tool to gain direct access to the details of the velocity autocorrelation function^53^. The same idea was exploited in Refs.^49–51^, using position correlation function rather than velocity, i.e. the Eq. (13).

Let us plug the anomalous velocity correlation function (19) into the Eq. (9). The result is

23 $$\begin{aligned} \ln \frac{S(\Delta )}{S(0)} = - \frac{\gamma ^2g^2D_{\alpha }}{(\alpha +1)(\alpha +2)}\biggl [ (\Delta +\delta )^{\alpha +2}+(\Delta -\delta )^{\alpha +2}-2\Delta ^{\alpha +2}-2\delta ^{\alpha +2} \biggr ], \end{aligned}$$

and, as expected^49^, it corresponds to that obtained using the position correlation function such as in Eq. (13) (see SOM). However, it does not correspond to the expression furnished in Ref.^53^, although a similar approach was implemented. Furthermore, in Ref.^52^ the NPG limit, $\delta \ll \Delta$, of (23) was derived: in this case the Eq. (4) corresponds to the Fourier transform of the MP.

In the SOM it is shown that the two last integrals appearing in Eq. (12) can be safely neglected for pulses interspacing $\Delta \gtrsim 2\delta$, leading to the more clear and manageable formula

24 $$\begin{aligned} \ln \frac{S(\Delta )}{S(0)} \simeq -\gamma ^2g^2\delta ^2D_{\alpha }\left[ \Delta ^{\alpha }-\frac{2\delta ^\alpha }{(\alpha +1)(\alpha +2)}\right] . \end{aligned}$$

The previous equation constitutes one of the central results of our work, as it can be considered the natural extension of the celebrated Stejskal–Tanner expression to the domain of anomalous diffusion. Notice, indeed, how the Stejskal–Tanner relation is recovered in case of Brownian diffusion, i.e. $\alpha =1$.

We will make use of Eq. (24) to fit synthetic NMR signals from anomalous diffusing systems.

### Fractional Brownian motion

The Fractional Brownian motion (FBM) represents a paradigmatic model for systems satisfying the hypothesis (6) and (7) and displaying anomalous diffusion on the score of persistent non-Markovian effects^65^. Moreover, being a Gaussian stationary process, it satisfies the hypotheses (6)–(7). The stochastic equation for the velocity is

25 $$\begin{aligned} v(t)=\xi _{FBM}(t), \end{aligned}$$

where the fractional Gaussian noise satisfies $\langle \xi _{FBM}(t)\rangle =0$ and $\langle \xi _{FBM}(t)\xi _{FBM}(t')\rangle =H(2H-1)K\left| t-t'\right| ^{2H-2}$. *K* is a positive constant, *H* is the Hurst exponent, $0<H<1$ and a comparison with Eq. (19) gives $\alpha =2H$ and $D_\alpha =\frac{K}{2}$.

We have generated synthetic DW-NMR signals from large ensembles of stochastic FBM trajectories, for different values of *K*, *H*, $\delta$ and *g*. The procedure used is detailed in the “Methods” section. Furthermore, we have fitted the numerical curves with the anomalous diffusion formula (24), in order to test the reliability and the robustness of our theoretical framework. The results are displayed in Fig. 2b and Supplementary Fig.S6 (see SOM) and reported in Table 2, where the discrepancy between the couple of $\alpha$ and $D_\alpha$ fitted and those implemented in the simulations is shown to be $\le 5\%$.

**Table 2:** Fit parameters of the synthetic curves obtained from FBM trajectories.

$\delta$ (s)	*g* (T/m)	Exact $D_\alpha =\frac{K}{2} ({\text {m}}^2/{\text {s}}^{\alpha }]$	Fitted $D_\alpha ({\text {m}}^2/{\text {s}}^{\alpha }]$	Exact $\alpha =2H$	Fitted $\alpha$	$D_\alpha$ error $(\%)$	$\alpha$ error $(\%)$
0.001	0.01	$2\times 10^{-9}$	$1.96133\times 10^{-9} \pm 11.7\times 10^{-13}$	0.5	$0.477207 \pm 3.45\times 10^{-4}$	$1.93\%$	$4.56\%$
0.02	0.01	$2\times 10^{-9}$	$1.99011\times 10^{-9} \pm 7.5\times 10^{-13}$	0.5	$0.496056 \pm 3.40\times 10^{-4}$	$0.80\%$	$1.65\%$
0.02	0.02	$2\times 10^{-9}$	$2.08086\times 10^{-9} \pm 24.57\times 10^{-12}$	0.5	$0.519624 \pm 7.01 \times 10^{-4}$	$3.92\%$	$3.09\%$
0.005	0.04	$2\times 10^{-9}$	$2.01488\times 10^{-9} \pm 5.6\times 10^{-13}$	0.5	$0.500618 \pm 3.12 \times 10^{-4}$	$1.14\%$	$4.02\%$
0.03	0.01	$2\times 10^{-9}$	$1.98559\times 10^{-9} \pm 2.29\times 10^{-12}$	0.7	$0.699299 \pm 6.80 \times 10^{-4}$	$0.72\%$	$0.10\%$
0.02	0.01	$1.5\times 10^{-9}$	$1.48289\times 10^{-9} \pm 3.1\times 10^{-13}$	1.2	$1.194670 \pm 1.07 \times 10^{-4}$	$1.14\%$	$0.44\%$
0.005	0.04	$1.5\times 10^{-9}$	$1.48345\times 10^{-9} \pm 1.4\times 10^{-13}$	1.2	$1.194503 \pm 9.3 \times 10^{-5}$	$0.57\%$	$0.14\%$
0.03	0.01	$8\times 10^{-10}$	$8.0741\times 10^{-10} \pm 4.2\times 10^{-13}$	0.8	$0.805499 \pm 2.91 \times 10^{-4}$	$0.93\%$	$0.69\%$
0.005	0.01	$8\times 10^{-10}$	$8.0080\times 10^{-10} \pm 4\times 10^{-14}$	0.7	$0.698734 \pm 4.9 \times 10^{-5}$	$0.10\%$	$0.18\%$
0.003	0.01	$1.5\times 10^{-9}$	$1.50691\times 10^{-9} \pm 1.3\times 10^{-13}$	0.7	$0.701762 \pm 9.1 \times 10^{-5}$	$0.46\%$	$0.25\%$

## Checking the assumptions: model’s discriminative power

In this section we test the discriminative power of our model, namely, the capability to discern processes that may diffuse normal or anomalous, but do not meet the criteria in Eqs. (6) and (7). It is crucial, indeed, that the use of the formula (22) and (24) is limited to Gaussian processes with stationary increments, otherwise the values of the fitted parameters may turn out to be biased and/or erroneous. We check this hypothesis by simulating NMR attenuation signals from stochastic systems that violate one or both the requirements (6) and (7).

**Figure 3:**
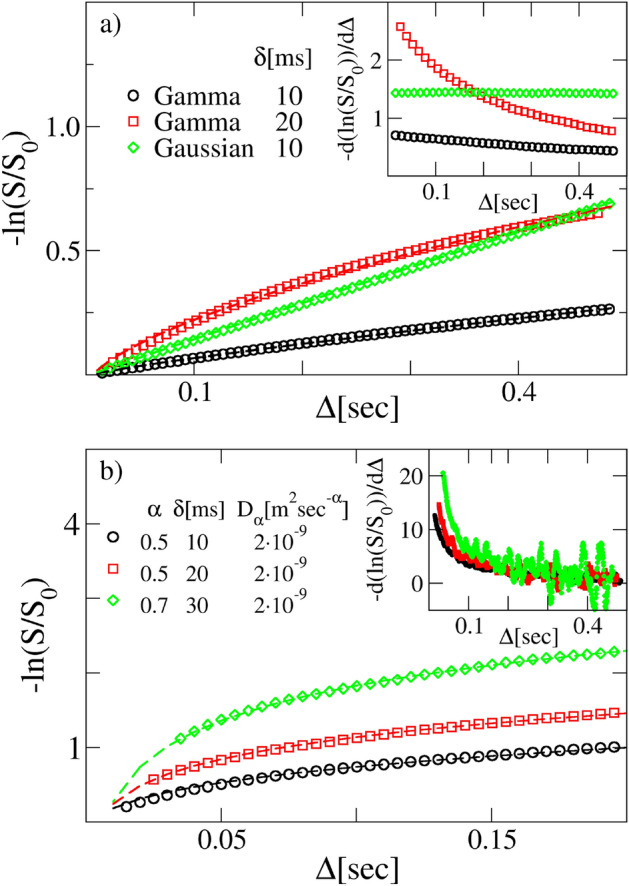
Panel (**a**) Superstatistical echo amplitudes. Main panel: comparison between different synthetic NMR signals obtained from SS trajectories (symbols) with a Gamma ($\theta =2 \times 10^{-9} \; {\text{m}}^2 \;{\text{s}}^{-1}$ and $k=0.5$) and a Gaussian ($D_*=2 \times 10^{-9} \; {\text{m}}^2 \; {\text{s}}^{-1}$ and $\sigma _D=10^{-10} \; {\text{m}}^2 \; {\text{s}}^{-1}$). PDFs and fitting curves obtained from Eq. (24) (dashed curves). The signals are obtained with a gradient $g=0.01$ T/m. Inset: trend of the derivative of the logarithm of the normalized DW-NMR signals shown in the main plot. Panel (**b**) CTRW echo amplitudes. Main plot: comparison between different synthetic NMR signals obtained from CTRW trajectories (symbols) and fitting curves obtained from Eq. (24) (dashed curves). The signals are obtained with a gradient $g=0.02$ T/m. Inset: trend of the derivative of the logarithm of the normalized NMR signals shown in the main plot.

The first case of study is the Brownian yet non-Gaussian diffusion. This expression tends to comprehend a large class of biological, soft, and active matter systems that exhibit normal diffusive dynamics with a non-Gaussian distribution of increments, thus violating the hypothesis (7). Several mathematical models have been introduced to reproduce this peculiar diffusion dynamics, whose most prominent examples are certainly the superstatistical BM^66^ and the diffusing diffusivities model^79,80^. In our study we implement the numerical simulation of stochastic trajectories based on the superstatistical (SS) model. This consists in integrating an overdamped version of the Langevin equation (20), i.e.

26 $$\begin{aligned} v(t)=\xi _{SS}(t), \end{aligned}$$

with $\langle \xi _{SS}\rangle =0$, $\langle \xi _{SS}(t)\xi _{SS}(t')\rangle =2D\delta (t-t')$ and *D* the diffusion coefficient. However the value of *D* is not constant for all trajectories, but it can assume any value drawn from a specific distribution *P*(*D*). We chose two types of *P*(*D*): a Gamma and a Gaussian distribution, the first with a scale parameter $\theta =2\times 10^{-9} \; {\text{m}}^2\; {\text{s}}^{-1}$ and shape parameter $k=0.5, 1.5$ and the second with mean $D_*=2\times 10^{-9} \; {\text{m}}^2 \; {\text{s}}^{-1}$ and standard deviation $\sigma _D=10^{-10} \; {\text{m}}^2 \; {\text{s}}^{-1}$. In the “Methods” section we clarify the numerical details of our procedure for generating the NMR signals from the stochastic trajectories achieved through the SS model. The results are shown in Fig. 3a. The first striking observation is that the signal does not show a linear trend, as instead one would expect for Brownian diffusing physical systems. In particular this appears clear when *P*(*D*) is a Gamma distribution function. Hence one could erroneously conclude that the relevant formula to use is the (24), with a complete misguided interpretation of the anomalous nature of the stochastic process (see Table 3). Additionally, when the linear behavior is respected, as in the case of *P*(*D*) Gaussian, the fitted value of *D* is close enough to the mean $D_*$ (as long as $\sigma _D$ is small), thus leading to the wrong conviction that the process is Brownian (see Table 2).

To substantiate further this finding, we have simulated another type of non-Gaussian linearly diffusing system, i.e. the Variance Gamma process, obtaining the same erroneous conclusions. This is plainly described in the SOM (see also Supplementary Fig. S5 and Table 5).

**Table 3:** Fit parameters of the synthetic curves obtained from SS trajectories obtained using a Gamma distribution with $k=0.5$ and a Gaussian distribution with $\sigma _D=10^{-10} \text {m}^2/{\text {s}}$.

$\delta$ (s)	*g* (T/m)	$\langle D\rangle =k\theta ({\text {m}}^2/s)$	Fitted $D_\alpha ({\text {m}}^2/{\text {s}}^{\alpha })$	Exact $\alpha$	Fitted $\alpha$
0.01	0.01	$1\times 10^{-9}$	$7.01043\times 10^{-10} \pm 1.194\times 10^{-12}$	1.0	$0.84871 \pm 0.00161$
0.02	0.01	$1\times 10^{-9}$	$3.99161\times 10^{-10} \pm 1.204\times 10^{-12}$	1.0	$0.66751 \pm 0.00299$
0.003	0.01	$1\times 10^{-9}$	$7.04554\times 10^{-10} \pm 1.440\times 10^{-12}$	1.0	$0.86612 \pm 0.00184$
0.005	0.01	$1\times 10^{-9}$	$8.96327\times 10^{-10} \pm 6.18\times 10^{-13}$	1.0	$0.93906 \pm 0.00066$
$\delta$ (s)	*g* (T/m)	$D_*({\text {m}}^2/{\text {s}}^{\alpha }]$	Fitted $D_\alpha ({\text {m}}^2/{\text {s}}^{\alpha }]$	Exact $\alpha$	Fitted $\alpha$
0.01	0.01	$2\times 10^{-9}$	$2.002813\times 10^{-9} \pm 2.35\times 10^{-13}$	1.0	$0.99741 \pm 0.00011$

**Table 4:** Fit parameters of the synthetic curves obtained from VGP trajectories.

$\delta$ (s)	*g* (T/m)	Exact $D=\frac{\sigma ^2}{2} ({\text {m}}^2/{\text {s}}]$	Fitted $D({\text {m}}^2/{\text {s}})$	*D* error $(\%)$	Fitted $\zeta ({\text {s}}^{-1}]$
0.01	0.02	$2\times 10^{-9}$	$6.7167\times 10^{-10} \pm 3.1\times 10^{-13}$	$66.41\%$	$7.1\times 10^5 \pm 1.2\times 10^8$
0.02	0.02	$2\times 10^{-9}$	$2.8797\times 10^{-10} \pm 3.9\times 10^{-13}$	$85.60\%$	$3.5\times 10^5 \pm 8.3\times 10^7$
0.03	0.02	$2\times 10^{-9}$	$1.6233\times 10^{-10} \pm 4.3\times 10^{-13}$	$91.84\%$	$2.4\times 10^5 \pm 7.1\times 10^7$
0.03	0.02	$1.5\times 10^{-9}$	$1.5191\times 10^{-10} \pm 3.4\times 10^{-13}$	$89.87\%$	$2.4\times 10^5 \pm 6.1\times 10^7$
0.003	0.02	$2\times 10^{-9}$	$1.61876\times 10^{-9} \pm 5.8\times 10^{-13}$	$19.06\%$	$2.4\times 10^5 \pm 9.9\times 10^8$
0.003	0.02	$1.5\times 10^{-9}$	$1.29447\times 10^{-9} \pm 1.6\times 10^{-13}$	$13.70\%$	$552\times 10^5 \pm 12$

The second case addressed concerns a model for anomalous diffusing systems which satisfies none of the hypotheses (6) and (7). The continuous-time random-walk (CTRW) model^36,46,81^ is a pure stochastic jump process, with jumps and waiting times that are uncorrelated and Markovian. For this system an equation like Eq. (2) can not be drawn and, therefore, a velocity does not have any meaning. In the “Methods” section it is plainly illustrated the numerical method to generate CTRW stochastic subdiffusive trajectories. The reconstructed DW-NMR attenuations for several values of *g*, $\delta$, $\alpha$ and $D_\alpha$ are shown in Fig. 3b and Supplementary Fig.S7 (see SOM). In Table 5 the parameters enforced in the simulations and the corresponding fitted values upon the formula (24) are reported. As in the case of the SS previously discussed, the fitting curves (dashed lines in Fig. 3 and Supplementary Fig.S7) seem to describe very well the numerical data. However, the inferred values of the anomalous parameters are dramatically divergent from the correct ones (see the values reported in Table 5).

**Table 5:** Fit parameters of the synthetic curves obtained from CTRW trajectories.

$\delta$ (s)	*g* (T/m)	Exact $D_\alpha ({\text {m}}^2/{\text {s}}^{\alpha }]$	Fitted $D_\alpha [{\text {m}}^2/{\text {s}}^{\alpha }]$	Exact $\alpha$	Fitted $\alpha$	$D_\alpha$ error $(\%)$	$\alpha$ error $(\%)$
0.01	0.02	$2\times 10^{-9}$	$7.7751\times 10^{-10} \pm 6\times 10^{-14}$	0.5	$0.308098 \pm 1.90\times 10^{-4}$	$61.12\%$	$38.38\%$
0.02	0.02	$2\times 10^{-9}$	$4.3879\times 10^{-10} \pm 2.3\times 10^{-13}$	0.5	$0.114993 \pm 1.04 \times 10^{-4}$	$78.06\%$	$77\%$
0.03	0.02	$2\times 10^{-9}$	$4.9302635\times 10^{-7} \pm 4.6619492\times 10^{-7}$	0.7	$5.3\times 10^{-5} \pm 5 \times 10^{-5}$	$24551\%$	$100\%$
0.003	0.02	$2\times 10^{-9}$	$1.71476\times 10^{-9} \pm 1.6\times 10^{-13}$	0.5	$0.536484 \pm 1.0 \times 10^{-4}$	$14.26\%$	$7.30\%$
0.003	0.02	$2\times 10^{-9}$	$1.84380\times 10^{-9} \pm 1.0\times 10^{-13}$	0.7	$0.714850 \pm 5 \times 10^{-5}$	$7.81\%$	$2.12\%$

We can now assert that our initial hypothesis is correct: although the fit appears to work well, the estimated parameters are wrong. They are erroneous because they have been quantified using a parametric data fit function that is not relevant for the type of microscopic molecular diffusion scrutinized. As stressed at the beginning of this section, this observation is at the core of the question of the model discriminative power.

To illustrate this crucial point, let us take the experimentalist perspective. Imagine having either a linear or non-linear NMR attenuation signal like those displayed in Figs. 2, or 3, without any prior knowledge about the hidden microscopical mechanisms that led to it. Applying the fitting formula (22) or (24) would give an excellent agreement between the theoretical curve and the experimental data. However, owing to the examples discussed above, how can we trust the estimates of these parameters? How can we be sure that the formula we are adopting is relevant for the system under consideration? The answer is implicit in the expressions (22) and (24).

**Figure 4:**
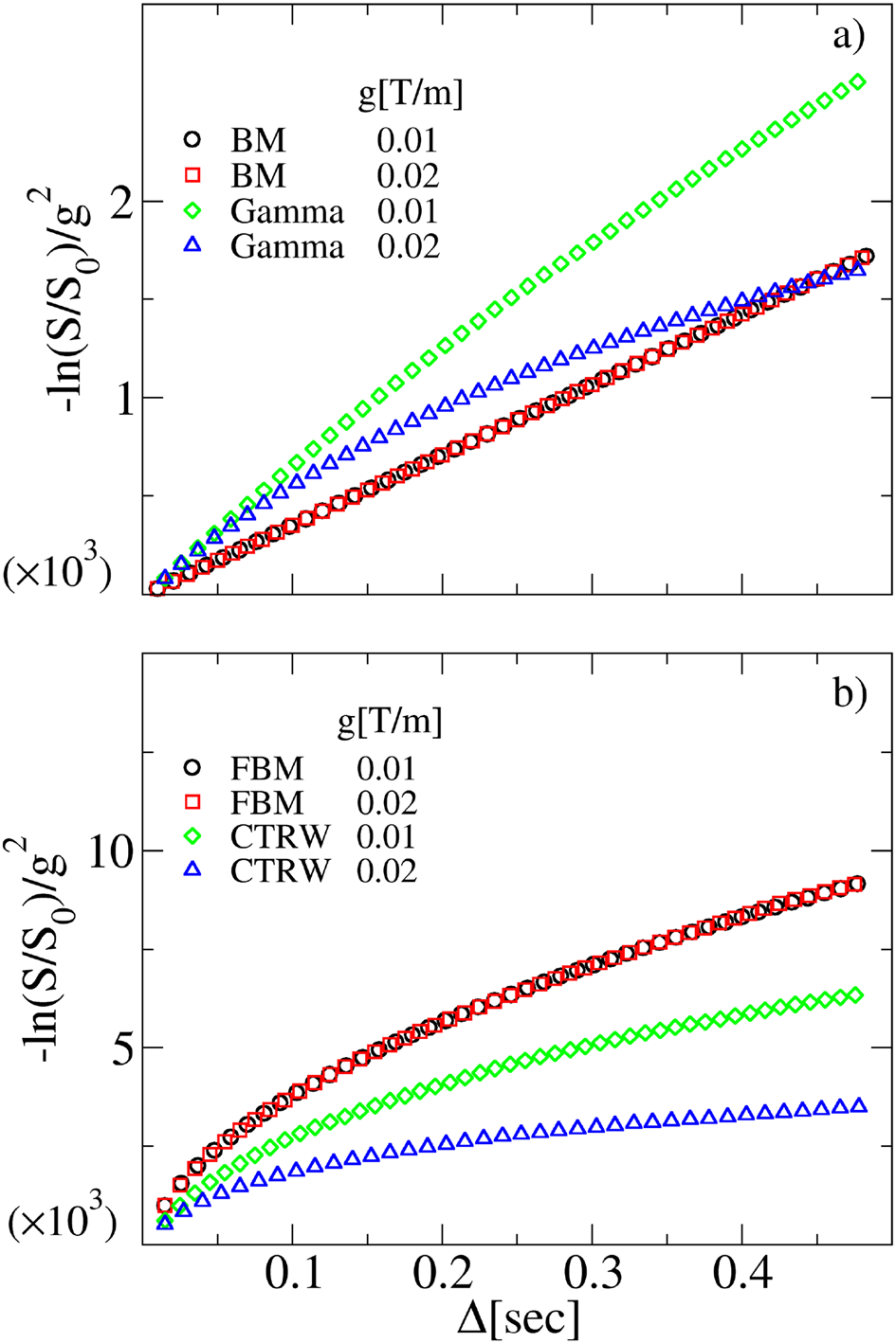
Model’s discriminative power. Panel (**a**) behavior of the logarithm of the normalized DW-NMR signal divided by $g^2$ as a function of $\Delta$, obtained from synthetic NMR signals of BM and SS model at two different *g*. We used a Gamma distribution as *P*(*D*) with $\theta =2\times 10^{-9}\; {\text {m}}^2~{\text {s}}^{-1}$ and $k=0.5$. The other parameters are: $D=2\times 10^{-9} \; {\text {m}}^2~{\text {s}}^{-1}$ and $\delta =0.01$ s. Panel (**b**) behavior of the same quantity as in panel (**a**), obtained from synthetic NMR signals of FBM and CTRW with $\alpha =0.5$, $D_{\alpha }=2\times 10^{-9}\; {\text {m}}^2~{\text {s}}^{-\alpha }$ and $\delta =0.01$ s. The different gradient rescaling of the curves is apparent and it is due to the fact that BM and FBM are based on Gaussian processes satisfying (6) and (7), while SS and CTRW are not.

At first instance, let us suppose to perform a couple of distinct experiments characterized by two gradient fields $g_1$ and $g_2$, keeping the value of $\delta$ unvaried. If the ensuing NMR attenuations $\ln \frac{S_1(\Delta )}{S_1(0)}$ and $\ln \frac{S_2(\Delta )}{S_2(0)}$ are of the form (22) or (24), namely the experimental system satisfies the hypothesis (6) and (7), therefore the rescaled functions $\frac{1}{g_1^2}\ln \frac{S_1(\Delta )}{S_1(0)}$ and $\frac{1}{g_2^2}\ln \frac{S_2(\Delta )}{S_2(0)}$ should collapse on top of each other. In the opposite case, the rescaled signals would appear well separated. This is indeed what can be seen in Fig. 4. Gaussian processes with stationary increments, such as BM of FBM, exhibit the expected collapse, while attenuations arising from SS or CTRW processes do not. This finding clearly evidences that fourth (kurtosis) and higher cumulants cannot be neglected in the expansion (5).

**Figure 5:**
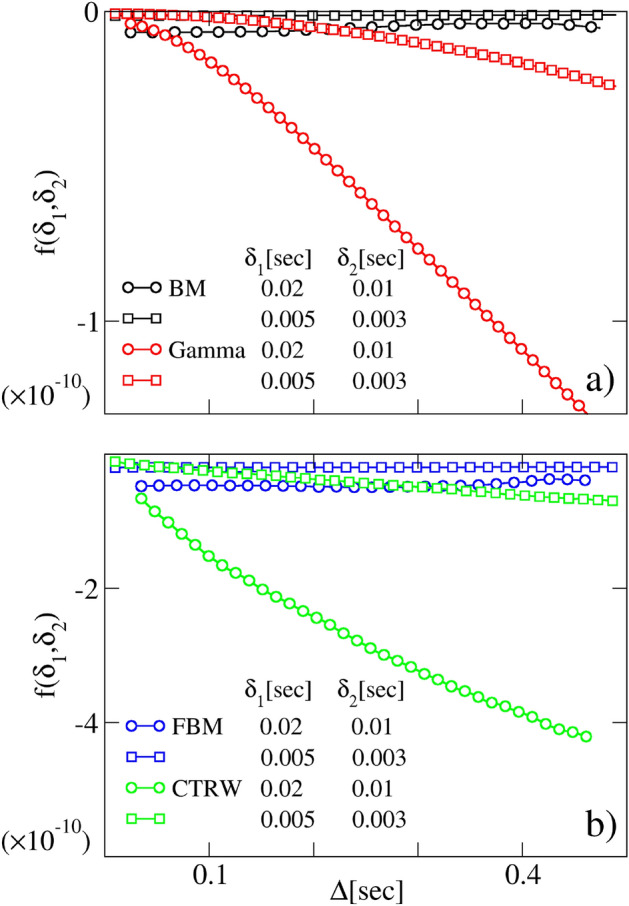
Model’s discriminative power. Panel (**a**) behavior of the $f(\delta _1,\delta _2)$ Eq. (27) as a function of $\Delta$, obtained from synthetic DW-NMR signals of BM and SS at different $\delta$, $D=2\times 10^{-9} \; {\text {m}}^2~{\text {s}}^{-1}$ and $g=0.01$ T/m. We used a Gamma distribution as PDF with $\theta =2\times 10^{-9} \; {\text {m}}^2~{\text {s}}^{-1}$ and $k=0.5$. Panel (**b**) behavior of the same quantity as in panel (**a**), obtained from synthetic DW-NMR signals of FBM and CTRW with $\alpha =0.5$, $D_{\alpha }=2\times 10^{-9} \; {\text {m}}^2~{\text {s}}^{-\alpha }$ and the same *g* as in panel (**a**) . The deviation from the Gaussian stationary signal is well visible.

Let us now consider the case of two experiments conducted using the same value of *g*, but with two different pulses $\delta _1$ and $\delta _2$. By rescaling the NMR attenuation signals as $\frac{1}{\delta ^2}\ln \frac{S(\Delta )}{S(0)}$, one immediately sees that the function

27 $$\begin{aligned} f(\delta _1,\delta _2)= (\gamma g)^{-2}\left[ \frac{1}{\delta _1^2}\ln \frac{S_1(\Delta )}{S_1(0)}-\frac{1}{\delta _2^2}\ln \frac{S_2(\Delta )}{S_2(0)}\right] \end{aligned}$$

must be independent of $\Delta$ for Gaussian processes with stationary increments. This is exactly what we found when analyzing the BM or FBM curves as in Fig. 5. The situation, however, changes considerably when we analyze DW-NMR signals coming from non-Gaussian processes like SS or CTRW. In this case, the quantity (27) exhibits a non-linear dependence on $\Delta$. This gives a sharp criterion to distinguish Gaussian processes with stationary increments from any other, where either one of the hypothesis (6)–(7) is violated.

To test effect of the experimental noise on the practicability of these validation rules, we added a Rician noise^82^ to the FBM and CTRW synthetic signals reported in Fig. 4. The results are displayed in Supplementary Fig. S9: the FBM collapse holds up to a value of $\Delta ^*$ depending on the signal-to-noise ratio (SNR), i.e. larger the noise, lower is the collapse bound $\Delta ^*$. Conversely, CTRW curves appear well separated also in the presence of strong SNR.

We stress that the methodological questions raised in this section have a validity that extends beyond the PFG-type of experiments. The MGSE method, for example, has been developed under the same assumptions of Gaussianity and stationarity of increments^18,68,70,71,75^. Hence, before applying any fitting formula derived according to a precise model of molecular diffusion, a good practice would be to ascertain whether both such a model and the physical process generating the DW-NMR signals, fulfil the hypothesis (6) and (7). By instance, in Ref.^32^ the subdiffusion of tetrafluoromethane inside the AlPO$_4$-5 zeolite channels was detected and analyzed. The formula (22) was used to characterize the self-diffusion molecular MSD arising from the DW-NMR signal, assuming the validity of the NPG approximation. This was possible thanks to the fact that the single-file model was rightfully considered the physical model relevant for the tetrafluoromethane diffusion inside the zeolite channels. As a matter of fact, molecular dynamics in single-file systems is known to be a clear example of FBM^74,83^. Yet, in Ref.^33^ evidence of anomalous diffusion, and transitional behavior among two distinct regimes, was provided for the segmental displacement in a monodisperse polystyrene polymer solution. Also in this case, within the NPG approximation, the formula used was (22), as the models considered for the polymer dynamics, were both giving rise to FBM, i.e. the Schweizer generalized Langevin equation model^84^, and the Doi–Edwards–deGennes model for reptation^74,85,86^.

## PFG NMR signal for the superstatistical model for Brownian yet non-Gaussian diffusion

The rescaling procedures of the DW-NMR signals define, without a doubt, whether the subtending stochastic process is Gaussian and with stationary increments or it is not. However, the case of signal rescaling failure does not reveal the nature of the process yielding to it, neither the correct formula to use to fit. As a matter of fact, any model of diffusion generates a different fitting formula which stems from the Eq. (4). In general, devising this expression is not an easy task.

The case of the SS model for Brownian yet non-Gaussian diffusion represents an exception. This case is treatable because the arising signal is just the Stejskal–Tanner formula, averaged over the diffusion distribution function *P*(*D*). The way the DW-NMR formula for SS model are derived is straightforward and is reported in the “Methods” section. We hereby report the fitting formula in case of Gamma and Gaussian *P*(*D*) respectively. The DW-NMR for Gamma distributed diffusion coefficient is given by

28 $$\begin{aligned} \begin{aligned} \ln \frac{S(\Delta )}{S(0)}= -k \ln (1+b\theta ), \end{aligned} \end{aligned}$$

where *k* and $\theta$ are the shape and scale parameters identifying the Gamma distribution function, and $b = \gamma ^2 g^2 \delta ^2 \Big (\Delta -\frac{\delta }{3}\Big )$ is the usual *b* value^6^.

When *P*(*D*) is a Gaussian distribution function of mean $D_*$ and standard deviation $\sigma _D$, the attenuation signal becomes

29 $$\begin{aligned} \ln \frac{S(\Delta )}{S(0)}= \Big ( -D_*b+\frac{1}{2}a^2D_*^2b^2 \Big )+ \ln \Big [ erfc\Big (\ -\frac{1}{\sqrt{2}a}+\frac{aD_*b}{\sqrt{2}}\Big ) \Big ] - \ln {\Big [erfc\Big (\ -\frac{1}{\sqrt{2}a}\Big )\Big ]}, \end{aligned}$$

where $a = \sigma _D/D_*$ and *erfc*(*x*) is the complementary error function^30^.

**Figure 6:**
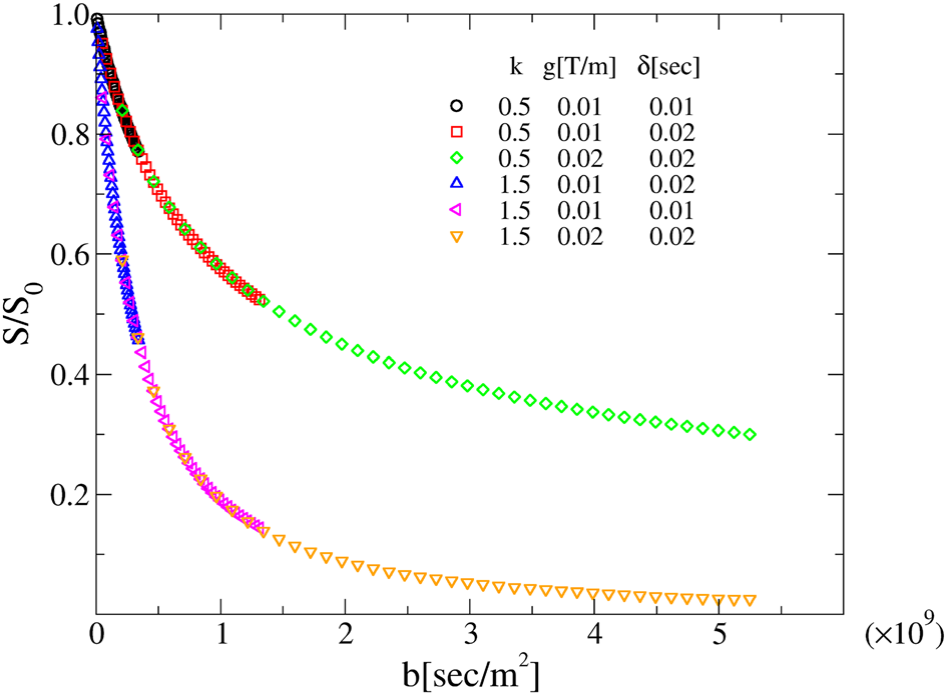
SS assessment criterion. Behavior of the synthetic rescaled DW-NMR signals as a function of the parameter $b= (\gamma g \delta )^2(\Delta -\delta /3)$ for SS systems with a Gamma distribution *P*(*D*).

The fitting formula Eqs. (28) and (29) show that the DW-NMR signals obtained from SS models depend uniquely on the *b* value. Interestingly, this property holds for any choice of *P*(*D*), different from (28) or (29). This means that, once plotted against *b*, signals obtained from different experiments collapse on top of each other, as indeed shown in Fig. 6. Once again, the experimentalist point of view comes to our aid. If a number of attenuation signals exhibit the linear or non-linear trends in Fig. 3a and fail the checks in Fig. 4a, their collapse as a function of *b* ensures that the are generated by Brownian yet non-Gaussian processes. Therefore the correct formula to be used are (28), (29) or any other expression drawn from the *P*(*D*) according to the very same procedure outlined in the “Methods” section. For the sake of completeness, in Supplementary Fig.S5 we show that DW-NMR signals obtained from CTRW do not show any collapse as a function of *b*.

## Validation rules: a practical example

**Figure 7:**
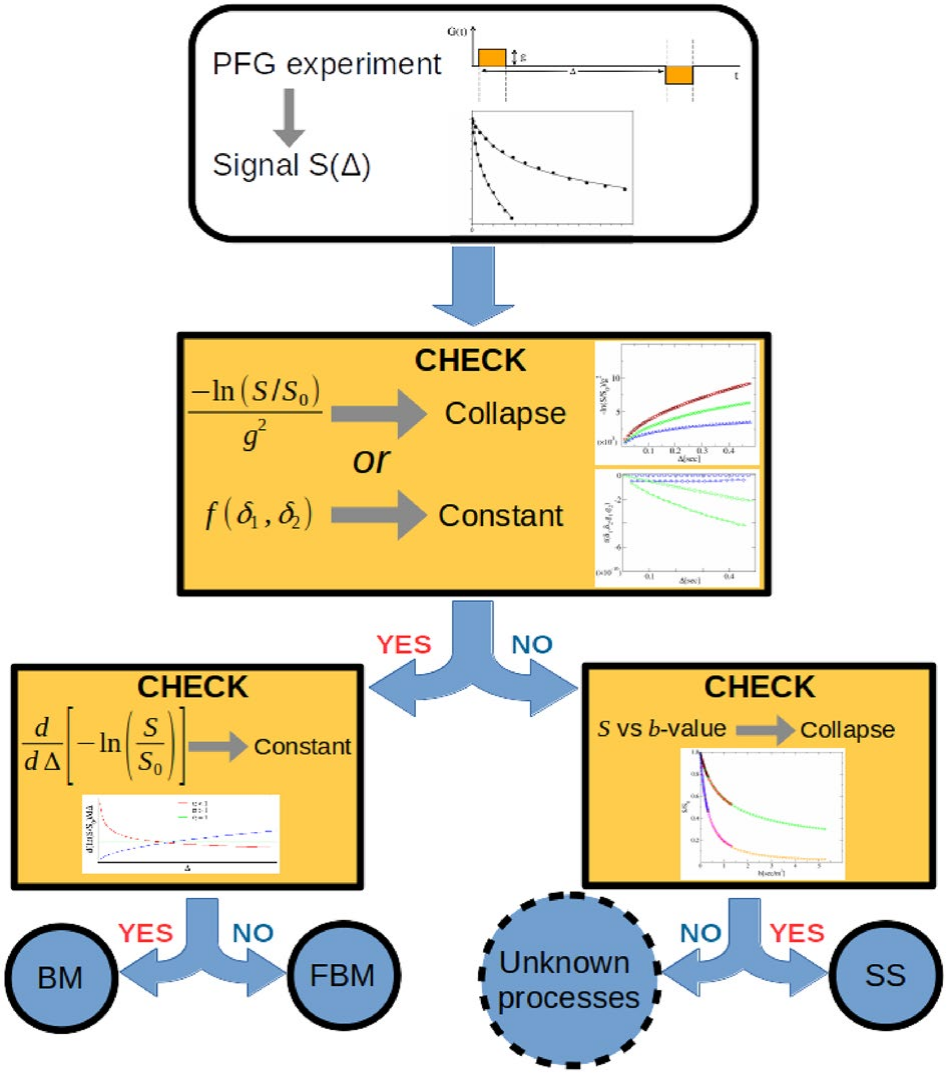
Flowchart of validation rules. The sequence of validation rules are summarized in this flowchart.

The set of checks outlined in the last two sections helps to disclose the nature of the molecular diffusion process yielding the DW-NMR signal, and to determine the pertinent parametric function to be used for its analysis. We hereby summarize them with the help of the flowchart in Fig. 7.

First, the NMR attenuations achieved at different experimental conditions (two distinct PFG pulses duration $\delta _1$ and $\delta _2$, or two distinct gradient strengths $g_1$ and $g_2$) must be rescaled as in Figs. 4 or 5. In case of curves collapse, the derivative of the signal must be performed. If the derivative is constant, the parametric function to use is that reported in Eq. (22), in the opposite case the anomalous expression reported in Eq. (24) applies. If the NMR signals rescaled according to Figs. 4 or 5 do not collapse on top of each other, they can be plotted as a function of *b* as in Fig. 6. If they show a satisfactory collapse, the formula (28) or (29) are the correct parametric functions for fitting the experimental PFG data. Otherwise, new formulas valid for different (anomalous) diffusion processes, accompanied by new validation rules, must be devised. For instance, the validation rules for CTRW-like processes will be the subject of a forthcoming investigation.

**Figure 8:**
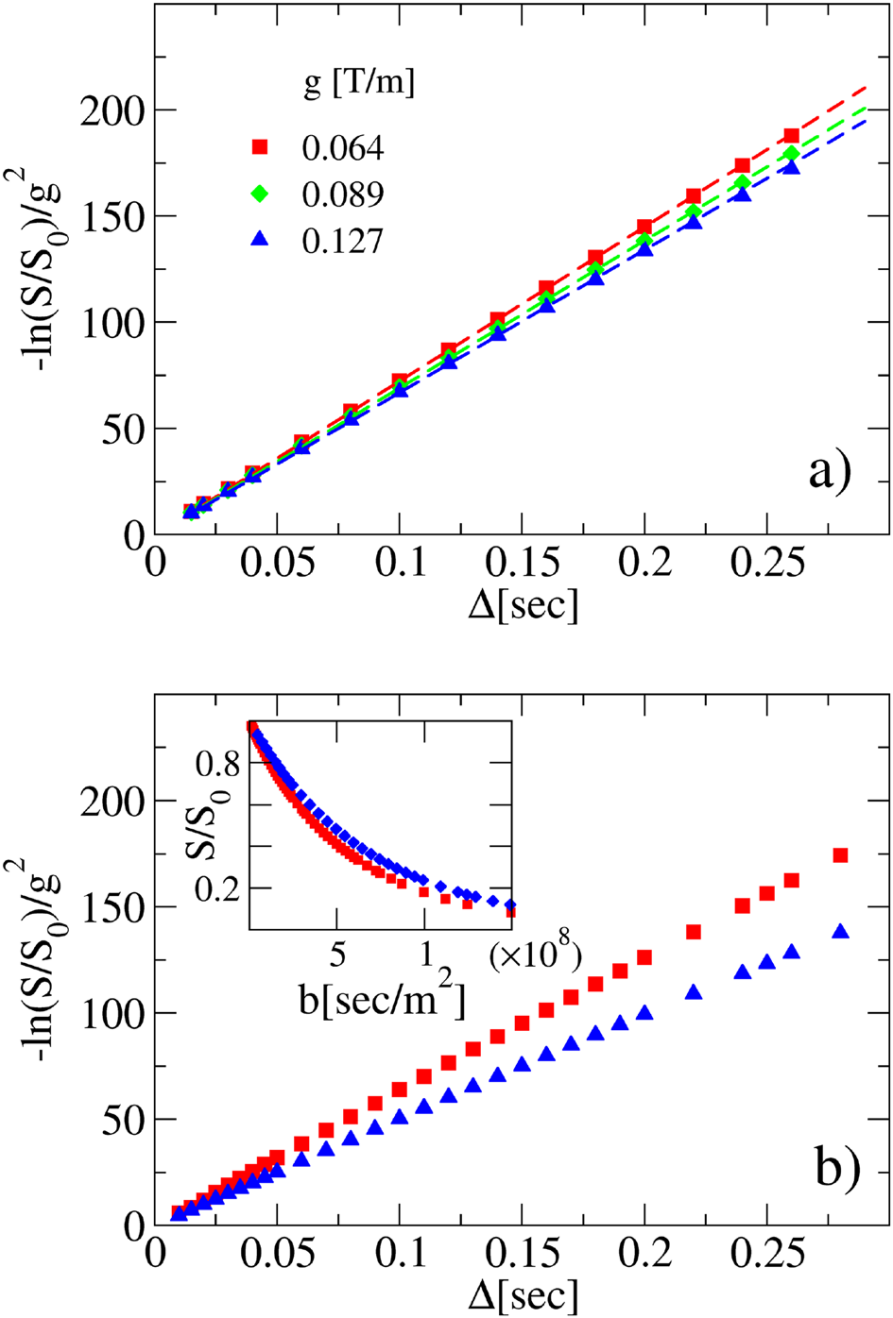
Experimental curve rescaling. DW-NMR signals of water diffusion coming from two different experiments are rescaled according to Fig. 5 (see “Methods”). Panel (**a**) diffusion of water inside water. The rescaled curve obtained at different values of the pulse field gradient strengths *g*, makes it possible to use the Stejskal–Tanner formula (22). The fitted values of *D* are $D=2.1\times 10^{-9} \; {\text {m}}^2/{\text {s}}$ for $g=0.064$ T/m, $D=2.0\times 10^{-9} {\text {m}}^2/{\text {s}}$ for $g=0.089$ T/m and $D=2.1\times 10^{-9} {\text {m}}^2/{\text {s}}$ for $g=0.127$ T/m with an error of about $10^{-12}$ in all cases. The $\zeta$ values are of order of $10^5$, meaning that the NPG approximation holds in this case $\delta \zeta \gg 1$. Panel (**b**) DW-NMR attenuation signals of water molecules diffusing in samples filled up with polystyrene microbeads mixture with nominal average diameters of 6, 10, 40 $\upmu$m^37,39^. Symbols refer to the values of *g* in Panel (**a**). The lack of collapse between the rescaled curves does not allow the use of the anomalous expression (24) for fitting. Inset: the same DW-NMR attenuation signals of panel (**b**) are plotted versus *b* value, showing a no solid rescaling.

Let us try the chain of validation rules on a practical example. In panel (a) of Fig. 8 three different signals of diffusing protons in free water are provided, obtained using a PFG-type acquisition sequence at various values of the gradient strength *g*. The rescaling of the curves as in Fig. 4 is respected and the derivative is constant. Hence the fit achieved through the use (22) is correct and the fitting parameters are reported inside the caption. Panel (b) shows the experimental DW-signal outcomes, hailing from water diffusion inside disordered heterogeneous systems composed by a mixture of 6 $\upmu$m, 10 $\upmu$m and 40 $\upmu$m polystyrene micro-beads with a 55$\%$ sphere packing^37,39^. In this case, the absence of collapse among the curves, achieved at different *g* after proper rescaling, highlights that the parametric fit function (24) cannot be used. Hence we try to replot the curves as a function of the *b* value (see the inset), but the collapse is not solid. We can conclude that the physical diffusive process giving rise to the signals in panel (b) is not even Brownian yet no-Gaussian. At this stage of our analysis we cannot conclude much more about the true nature of the process, neither about the right formula to fit the DW-NMR data. The details of both the experimental setups are provided in the “Methods” section.

## Conclusions

We have furnished a comprehensive theory of the diffusion NMR attenuation signals, under the Gaussian approximation in cumulant expansion. We have shown that PFG constitutes an excellent experimental method to probe the molecular velocity autocorrelation properties. This is particularly compelling for systems exhibiting persistent long-standing memory effects. Indeed, opposite to previous theories that made use of MGSE to infer the low-frequency part of the velocity spectrum^17,68,69^, we adopted the PFG sequences to ascertain the long time behavior of the velocity autocorrelation function. In particular, for power-law behavior such that in Eq. (19) we demonstrated that a non-linear decay of the logarithm of the signal is to be expected, corresponding to a stretched-exponential (KWW) attenuation. In turn, this represents the signature of anomalous diffusion, as compactly expressed in the Eq. (16).

When applied to BM, our theory provides the final expression for the Stejskal–Tanner formula, including the corrections due to the interplay between the three times scales characterizing the process, $\Delta$, $\delta$ and $\zeta ^{-1}$. Most importantly, in the case of anomalous diffusion, the fitting formula (24) is presented here for the first time, although similar approaches were proposed in the past. It represents an excellent tool for both the classification and the prediction of the PFG signal decay, as we have shown by testing it on synthetic signals from FBM trajectories. The same agreement is expected to hold for any Gaussian system with stationary increments, such as those governed by generalized Langevin equation, fractional Langevin equation or, in general, generalized fractional Langevin equation^87^. Moreover, we stress that our results are not only valid in the NPG limit, but they hold in any practical condition as well, such as those in clinical NMR scanners where the gradient pulse width $\delta$ of PFG is usually comparable to $\Delta$ duration. However, we did not restrict our analysis to the illustration of the model’s capability to describe Gaussian processes with stationary increments. A fundamental aspect of our theory consisted in the exact determination of the limits of its application, showing the potential of committing substantial errors in the diffusion parameters estimation, notwithstanding the apparent agreement between fitting parametric functions and DW-attenuation data. Flagrant evidence is furnished in Fig. 3a, where a process that is Brownian but not Gaussian, may generate stretched exponential echos amplitudes. Hence, it appears clear how the appraisal of the correct formula to use, and in which conditions it must be used, constitutes *the* essential question for the correct interpretation of the DW-NMR amplitudes. Therefore, it is of fundamental importance to provide the NMR scientist’s toolkit with the sequence of validation rules ready to be implemented. In this article we sketched the first ones, defining, at the same time, a *modus operandi* valid for the next.

The chain of validation rules constitutes, to our advice, the most efficient and costless way of determining the type of diffusion and the microscopic model for its correct interpretation. Our theoretical framework is entirely built on the analysis of the $\Delta$ dependence of the DW-NMR signal. Nothing prevents, however, to gain insights by probing its *g* dependence, as shown by checking the Gaussianity assumption by the $g^2$ rescaling. In future, we expect that validation rules for more complex diffusive scenario will involve both time and magnetic field dependence of *S*(*t*)/*S*(0). For instance, a viable validation rule for the appearance of the localization regime in bounded diffusion systems could be the rescaling of the attenuation signal by $g^{2/3}$^57,58,63,64^.

At last, we want to draw the reader’s attention to an innovative aspect of our analysis, somehow implicit in the previous discussion. The question of the correct choice of a microscopical model has been recognized to be a crucial issue, for systems displaying anomalous diffusion^88–90^. Different models may produce the law (18), although the mechanisms subtending the non-linear diffusion are completely different. This is the case, for instance, of FBM and CTRW as shown in Figs. 2b and 3b. Therefore, assessing the right model provides fundamental insights into the microscopical origins of the anomalous transport. Moreover, these considerations extends also to normal diffusing systems, that exhibit linear MSD although the MP is not Gaussian, i.e. the class of mathematical models which go under the name of Brownian yet non-Gaussian diffusion^66^. In our analysis we addressed the case of one of these models, i.e. the superstatistical model. So far, the only methods available to discern among (anomalous) diffusion models were limited to the realm of single particle tracking^89,91^, with the obvious drawback that a very large number of trajectories are needed to reach a satisfactory reliable assessment, and only in-vitro experiments can be conducted. In this study we have provided, for the first time, the theoretical evidence that the NMR using PFG sequences may constitute a very sensitive non-invasive tool, generating a well defined chain of validation rules sifting for the correct model of (anomalous) diffusion. This question is all the more timely since, in the last decade, with improved microscopy imaging and tracking methods, it became clear that a single trajectory exhibits spatial and temporal heterogeneity, and the picture of a constant anomalous exponent and/or a constant generalized diffusion coefficient is not tenable^92–95^. The complete characterization of echo amplitudes generated by non-Gaussian anomalous diffusing processes such as CTRW fractional motion (FM)^48^, or different models of Brownian yet no-Gaussian diffusion such as diffusing diffusivity models^79,80^, will be the subject of forthcoming publications. However, this study constitutes the first conceptual step toward the use of NMR as a experimental tool for characterizing different diffusive processes, and their microscopical origins.

## Methods

### Calculation of NMR PFG signal attenuation: velocity autocorrelation function

The derivation of Eqs. (22) and (23) is hereby sketched, the full theory is reported in SOM. First, we must calculate the time integral of $F(t)F(t-s)$ and its dependence on *s*. As *F*(*t*) is

30 $$\begin{aligned} F(t) = {\left\{ \begin{array}{ll} 0 &{} 0<t<t_1 \\ g(t-t_1) &{} t_1<t<t_1+\delta \\ g\delta &{} t_1+\delta<t<t_1+\Delta \\ g(t_1+\Delta +\delta -t) &{} t_1+\Delta<t<t_1+\Delta +\delta \\ 0 &{} t>t_1+\Delta +\delta , \end{array}\right. } \end{aligned}$$

it turns out that only few values of *s* guarantee that the product $F(t)F(t-s)$ is different than 0 (see the graphical representation in Supplementary Fig. S2 in the SOM). In particular the product vanishes for $s>\Delta +\delta$ and is non zero in four separate *s*-intervals $[0,\delta ]$, $[\delta ,\Delta -\delta ]$, $[\Delta -\delta ,\Delta ]$, $[\Delta ,\Delta +\delta ]$. Performing the integrals in each interval (see Supplementary Eqs. (S4)–(S7) in the SOM) we obtain the result (12). The Eq. (22) for BM can be derived integrating respect to *s* and using the velocity autocorrelation function $C(s)=k_BT e^{-\zeta s}$. In the same way the result in Eq. (23) can be obtained using $C(s)\sim \alpha (\alpha -1)D_\alpha s^{\alpha -2}$ and assuming $\delta$ large enough to ensure $\int _0^\delta ds\,C(s)\rightarrow \alpha D_\alpha \delta ^{\alpha -1}$ (see SOM).

### Monte Carlo simulations

#### BM

BM trajectories have been simulated integrating the Langevin equation with a time step $dt=10^{-3}$ s. We simulated an ensemble of $N_t=10^5$ trajectories for different values diffusion coefficient *D* reported in Table 1 of the SOM.

#### FBM

FBM trajectories have been simulated using the Davies–Harte method^96^ with a time step $dt=10^{-3}$ s. We simulated an ensemble of $N_t=10^5$ trajectories for different values of generalized diffusion coefficient $D_\alpha$ and anomalous exponent $\alpha$ reported in Table 2 of the SOM.

#### SS

SS trajectories have been simulated by the same protocol of the BM trajectories. In each trajectory we used a different diffusion coefficient *D* extracted from a probability density function *p*(*D*).

#### CTRW

We simulated the CTRW dynamics following the method in Ref.^97^; we generated the sequences of independent and identically distributed waiting times and jumps starting from two independent uniform random numbers $\in (0,1)$ and using the two transformations due to Chambers et al.^98^ and Kozubowski and Rachev^99^. The jump sequences thus obtained are characterized by a symmetric Lévy $\alpha$-stable probability density and a length parameter $\gamma _x$, while the waiting times sequences are determinated by the $\beta$ parameter of the Mittag-Leffler probability density and a time parameter $\gamma _t$. As we simulate only subdiffusive motion, the jumps are drawn from a Gaussian distribution. The $\gamma _x$ and $\gamma _t$ quantity are connected to the generalized diffusion coefficient $D_{\alpha }$ by the relation $D_\alpha =\gamma _x^{2}/[\gamma _t^{\beta }\Gamma (1+\beta )]$, where $\Gamma (x)$ is the gamma function. We performed $N_t=5\times 10^4$ CTRW Monte Carlo simulations using $D_\alpha =2\times 10^{-9}~{\text {m}}^2 \; {\text {s}}^{-1}$ and $\gamma _t=10^{-4}$ s. We sampled the trajectories with a time step $dt=10^{-5}$ s.

### Synthetic NMR signal

A way to obtain a synthetic NMR signal consists in simulating a representative set of trajectories $\textbf{r}(t)$, calculating the acquired dephasing $\phi (t)=\gamma \int _0^t dt' {\textbf{r}}(t')\cdot {\textbf{G}}(t')$ for each trajectory and obtaining the free induction decay (FID) $S/S(0)={\textbf{E}}\{e^{i\phi } \}$ by means of an average of the simulated outcomes^100^. In particular to create the NMR signal we utilized the PFG sequence^1^ with bipolar diffusion gradient pulses of constant amplitude *g* that are turned on during the time interval $[t_1, t_1+\delta ]$ and $[t_1+\Delta , t_1+\Delta +\delta ]$ (see Fig. 1 in the main text and Supplementary Fig. S1 in the SOM). We used $t_1=0.005$ s.

### PFG NMR signal for the superstatistical model of Brownian yet non-Gaussian diffusion

In systems with Brownian yet non-Gaussian diffusion the peculiar behavior emerges due to the fact that different particles, located in different environments, are characterized by different transport properties . Defining *p*(*D*) the distribution of the local diffusivities, the rescaled NMR signal is given by

31 $$\begin{aligned} \frac{S(t)}{S(0)}=\int _0^{\infty }\exp \Big [ -\gamma ^2 g^2 \delta ^2 D\Big (\Delta -\frac{\delta }{3}\Big )\Big ] p(D) dD. \end{aligned}$$

In particular if *p*(*D*), with $D\ge 0$, is a normalized Gamma distribution with parameters *k* and $\theta$ the above expression becomes

32 $$\begin{aligned} \begin{aligned} \frac{S(t)}{S(0)}=&\frac{1}{\Gamma (k)\theta ^k}\int _0^{\infty }\exp \Big [ -\gamma ^2 g^2 \delta ^2 D\Big (\Delta -\frac{\delta }{3}\Big )\Big ] D^{k-1}\exp \Big (-\frac{D}{\theta }\Big ) dD \\= &\frac{1}{\Gamma (k)\theta ^k}\int _0^{\infty } D^{k-1}\exp \Big [-D\Big (b+\frac{1}{\theta }\Big )\Big ] dD, \end{aligned} \end{aligned}$$

where $\Gamma (k)$ is the Gamma function and $b = \gamma ^2 g^2 \delta ^2 \Big (\Delta -\frac{\delta }{3}\Big )$.

Defining $u=D\Big (b+\frac{1}{\theta }\Big )$ we have

33 $$\begin{aligned} \begin{aligned} \frac{S(t)}{S(0)}= \frac{1}{\Gamma (k)\theta ^k}\int _0^{\infty } \frac{u^{k-1}e^{-u}}{\Big ( b+\frac{1}{\theta }\Big )^k } du, \end{aligned} \end{aligned}$$

and using the definition of the Gamma function we obtain

34 $$\begin{aligned} \begin{aligned} \frac{S(t)}{S(0)}= \frac{1}{\theta ^k} \frac{1}{\Big ( b+\frac{1}{\theta }\Big )^k }. \end{aligned} \end{aligned}$$

If, on the other hand, *p*(*D*), with $D\ge 0$, is a normalized Gaussian function with mean equal to $D_*$ and variance $\sigma _D$ the rescaled NMR signal is given by

35 $$\begin{aligned} \begin{aligned} \frac{S(t)}{S(0)}&=\sqrt{\frac{2}{\pi }}\frac{1}{\sigma _D\cdot erfc[-D_*/(\sqrt{2}\sigma _D)]}\int _0^{\infty }\exp \Big [ -\frac{(D-D_*)^2}{2\sigma _D^2}\Big ]\exp (-bD) dD \\&= \sqrt{\frac{2}{\pi }}\frac{1}{\sigma _D\cdot erfc[-D_*/(\sqrt{2}\sigma _D)]}\int _0^{\infty }\exp \Big [ -\frac{(D-D_*)^2+2\sigma _D^2 bD}{2\sigma _D^2}\Big ] dD \\&= \sqrt{\frac{2}{\pi }}\frac{1}{\sigma _D\cdot erfc[-D_*/(\sqrt{2}\sigma _D)]}\exp \Big [\frac{(D_*-\sigma _D^2 b)^2-D_*^2}{2\sigma _D^2}\int _0^{\infty }\exp \Big [ -\frac{(D-D_*+\sigma _D^2b)^2}{2\sigma _D^2}\Big ] dD, \end{aligned} \end{aligned}$$

with *erfc*(*x*) is the complementary error function. Defining $y=\frac{D-D_*+\sigma _d^2b}{\sigma _d}$ we have

36 $$\begin{aligned} \begin{aligned} \frac{S(t)}{S(0)}=&\sqrt{\frac{2}{\pi }}\frac{1}{erfc[-D_*/(\sqrt{2}\sigma _D)]}\exp \Big [\frac{(D_*-\sigma _D^2 b)^2-D_*^2}{2\sigma _D^2}\Big ]\int _{c}^{\infty }\exp \Big (-\frac{y^2}{2}\Big ) dy, \end{aligned} \end{aligned}$$

with $c=\frac{\sigma _D^2b-D_*}{\sigma _D}$. If $a = \sigma _D/D_*$ we have the final expression for the rescaled NMR signal

37 $$\begin{aligned} \begin{aligned} \frac{S(t)}{S(0)}=&\exp \Big (-D_*b+\frac{1}{2}a^2D_*^2b^2\Big )\frac{erfc\Big (\ -\frac{1}{\sqrt{2}a}+\frac{aD_*b}{\sqrt{2}}\Big )}{erfc[-1/(\sqrt{2}a)]}. \end{aligned} \end{aligned}$$

### Experimental methods

NMR diffusion measurements were performed on a Bruker Avance system, operating at 9.4 T with a micro-imaging probe (10 mm internal diameter bore) and equipped with a gradient unit characterized by maximum magnetic field gradient strength of 1.2 T/m , and a rise time of 100 $\upmu$s. The temperature of each sample was fixed at 291 K. A spectroscopic pulsed gradient stimulated echo (PGSTE)^101^ with $\delta$ = 4.4 ms and 2.2 ms, g = 0.064, 0.089 and 0.12 T/m along the x-axis, repetition time TR = 5000 ms, number of averaged signals NSA = 32, and 48 values of $\Delta$ in the range 10–1000 ms was used to collect data.

One sample was carried out using equal volume fractions of polystyrene micro-beads (Microbeads AS, Norway) with nominal average diameters of 40 $\upmu$m, 10 $\upmu$m, and 6 $\upmu$m mixed inside a 10 mm NMR tube filled up to a volume of approximately 2 cm$^3$ with a solution of polyoxyethylene-sorbitan-mono-laurate (Tween 20) $10^{-6}$ M and deionized water. The sample was investigated four months after its preparation. Moreover, one NMR tube filled up with free water was also analyzed with $\delta$ = 2.2 ms and g = 0.089 and 0.12 T/m along the x-axis .

## Supplementary Information

Supplementary Information.

## Data Availability

All data generated or analysed during this study are included in this published article (and its Supplementary Information files).
