# Supplementary material for: A new perspective of molecular diffusion by nuclear magnetic resonance
Source: Sci Rep. 2023 Jan 30;13:1703. doi: 10.1038/s41598-023-27389-7 (PMC9887074; doi:10.1038/s41598-023-27389-7)
Supplement: Supplementary file 1 — Supplementary Information. [file 41598_2023_27389_MOESM1_ESM.pdf]

Supplemental Material - A new perspective of molecular diffusion by Nuclear Magnetic Resonance

Giulio Costantini[1], Silvia Capuani[1], Francis Allen Farrelly[2], Alessandro Taloni[2]

[1]Istituto Sistemi Complessi, Consiglio Nazionale delle Ricerche, UOS Sapienza, 00185 Rome, Italy
[2]Istituto Sistemi Complessi, Consiglio Nazionale delle Ricerche, via dei Taurini 19, 00185 Rome, Italy

Contents

1 Calculation of NMR signal attenuation in pulsed field gradient (PFG) experiments: velocity autocorrelation function. 2

2 Calculation of NMR signal attenuation in PFG experiments: position autocorrelation function. 8

3 Variance gamma Process 11

4 Supplementary figures 14

5 Rician noise effect 16

1 Calculation of NMR signal attenuation in pulsed field gradient (PFG) experiments: velocity autocorrelation function.

The PFG $G(t)$ is shown in Fig.S1 (upper panel):

$$G(t) = \begin{cases} 0 & 0 < t < t_1 \\ g & t_1 < t < t_1 + \delta \\ 0 & t_1 + \delta < t < t_1 + \Delta \\ -g & t_1 + \Delta < t < t_1 + \Delta + \delta \\ 0 & t > t_1 + \Delta + \delta. \end{cases} \tag{S1}$$

The integral of the pulse gradient field is

$$F(t) = \begin{cases} 0 & 0 < t < t_1 \\ g(t - t_1) & t_1 < t < t_1 + \delta \\ g\delta & t_1 + \delta < t < t_1 + \Delta \\ g(t_1 + \Delta + \delta - t) & t_1 + \Delta < t < t_1 + \Delta + \delta \\ 0 & t > t_1 + \Delta + \delta \end{cases} \tag{S2}$$

and it is shown in Fig.S1 (lower panel).
The key quantity appearing in the Eq.(1) is

$$\tilde{I} = \int_0^{TE} F(t)F(t - s)dt, \tag{S3}$$

and must be evaluated summing the different contributions coming from the values $0 < s < TE$. We will do it with the help of Fig.S2. Here $F(t)$ and $F(t - s)$ are plotted as functions of the integration variable t. The non-zero contributions are

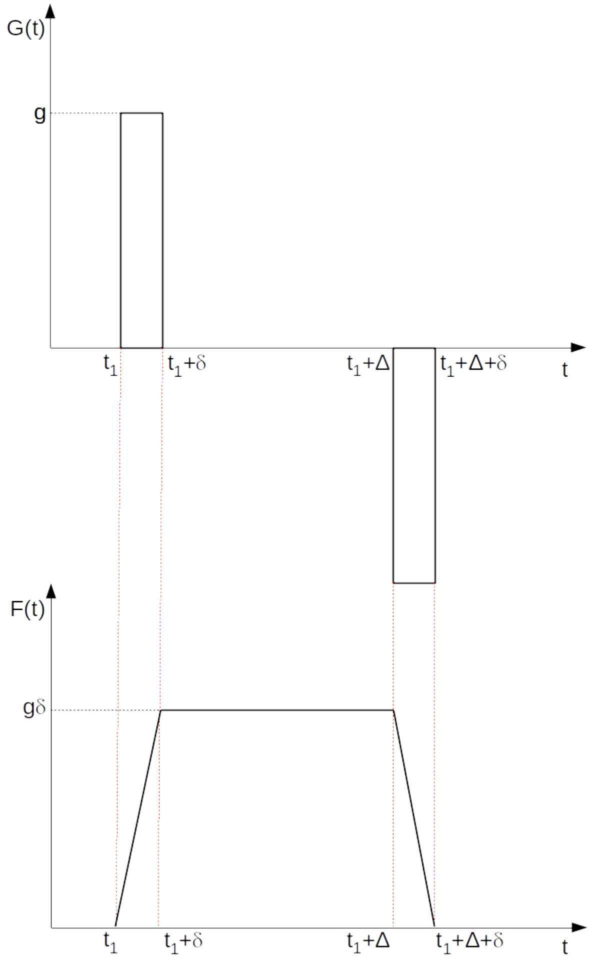

Figure S1: PFG sequence. The NMR pulse gradient sequence $G(t)$ is shown in the PFG formulation. The effective gradient is achieved without resorting the the intermediate π radio frequency pulse, but inverting the sign of the second pulse $(-g)$. In the bottom panel, the integral of the gradient field is shown: $F(t) = \int_0^t dt' G(t')$.

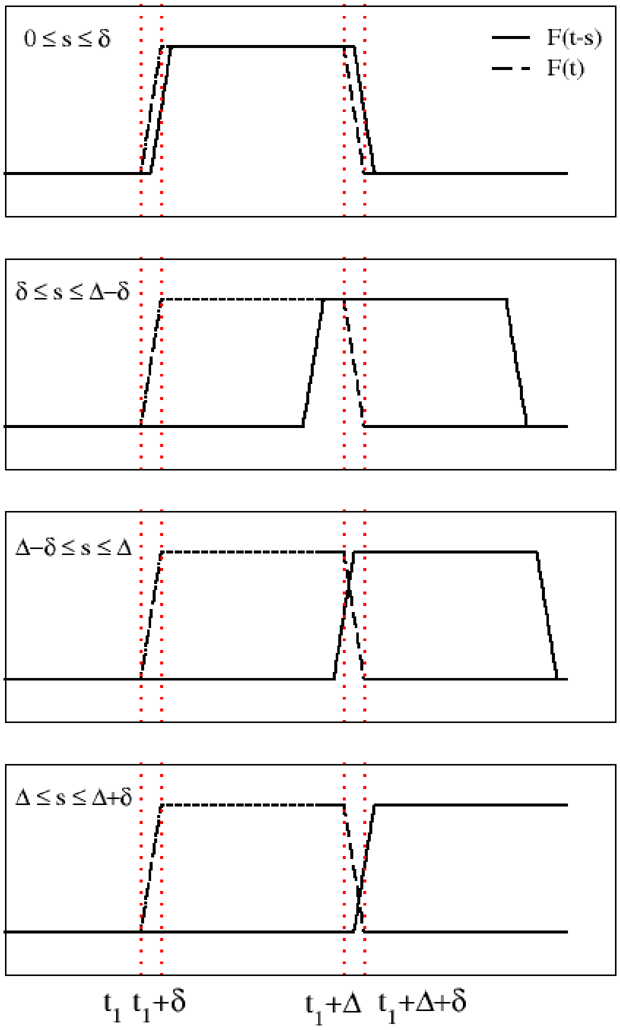

Figure S2: Graphical representation of the contributions in the Eq.(S3). The quantities $F(t)$ and $F(t-s)$ are shown for different values of s. The four panels correspond to the terms expressed in mathematical form in Eqs.(S4)-(S7).

- $0 \leq s \leq \delta$

$$\int_{t_1+s}^{t_1+\delta} F(t)F(t-s)dt + \int_{t_1+\delta}^{t_1+\delta+s} F(t)F(t-s)dt + \int_{t_1+\delta+s}^{t_1+\Delta} F(t)F(t-s)dt + \int_{t_1+\Delta}^{t_1+\Delta+s} F(t)F(t-s)dt + \int_{t_1+\Delta+s}^{t_1+\Delta+\delta} F(t)F(t-s)dt; \tag{S4}$$

- $\delta \leq s \leq \Delta - \delta$

$$\int_{t_1+s}^{t_1+\delta+s} F(t)F(t-s)dt + \int_{t_1+\delta+s}^{t_1+\Delta} F(t)F(t-s)dt + \int_{t_1+\Delta}^{t_1+\Delta+\delta} F(t)F(t-s)dt; \tag{S5}$$

- $\Delta - \delta \leq s \leq \Delta$

$$\int_{t_1+s}^{t_1+\Delta} F(t)F(t-s)dt + \int_{t_1+\Delta}^{t_1+\delta+s} F(t)F(t-s)dt + \int_{t_1+\delta+s}^{t_1+\Delta+\delta} F(t)F(t-s)dt; \tag{S6}$$

- $\Delta \leq s \leq \Delta + \delta$

$$\int_{t_1+s}^{t_1+\Delta+\delta} F(t)F(t-s)dt. \tag{S7}$$

Making use of the explicit form of $F(t)$ (S2) in the Eqs.(S4)-(S7) and performing the integration over s, we obtain

$$\begin{aligned}
&\int_0^\delta g^2\Big[\delta^2(\Delta - \frac{\delta}{3}) + s^2(\frac{s}{3} - \delta)\Big]C(s)ds + \\
&+ g^2\delta^2 \int_\delta^\Delta (\Delta - s)C(s)ds + \\
&+ \frac{g^2}{6}\int_{\Delta-\delta}^{\Delta} (\delta - \Delta + s)^3 C(s)ds + \\
&+ \frac{g^2}{6}\int_{\Delta}^{\Delta+\delta} (\delta + \Delta - s)^3 C(s)ds,
\end{aligned} \tag{S8}$$

confirming the result of Eq.(6).

Let us consider the Brownian motion and its velocity autocorrelation function $C(s) = k_B T e^{-\zeta s}$. The integrals in (S8) are easily evaluated:

$$\int_0^\delta ds\, C(s) \left(\Delta - \frac{\delta}{3}\right) = D\left(1 - e^{-\delta\zeta}\right)\left(\Delta - \frac{\delta}{3}\right); \tag{S9}$$

$$\int_\delta^\Delta ds\, C(s)\,(\Delta - s) = D\left[\frac{e^{-\Delta\zeta}}{\zeta} + e^{-\delta\zeta}\left(\Delta - \delta - \frac{1}{\zeta}\right)\right]; \tag{S10}$$

$$\int_0^\delta ds\, C(s) s^2 \left(\delta - \frac{s}{3}\right) = D\left[\frac{2}{\zeta^2}\left(\delta - \frac{1}{\zeta}\right) + e^{-\delta\zeta}\left(\frac{2}{\zeta^3} - \frac{\delta^2}{\zeta} - \frac{2\delta^3}{3}\right)\right]; \tag{S11}$$

$$\int_{\Delta-\delta}^\Delta ds\, \frac{C(s)}{6}\,(\Delta - \delta - s)^3 = \frac{D}{6} e^{-\Delta\zeta}\left[\delta^3 + \frac{3\delta^2}{\zeta} + \frac{6\delta}{\zeta^2} + \frac{6}{\zeta^3}\left(1 - e^{\delta\zeta}\right)\right]; \tag{S12}$$

$$\int_\Delta^{\Delta+\delta} ds\, \frac{C(s)}{6}\,(\Delta + \delta - s)^3 = \frac{D}{6} e^{-\Delta\zeta}\left[\delta^3 - \frac{3\delta^2}{\zeta} + \frac{6\delta}{\zeta^2} - \frac{6}{\zeta^3}\left(1 - e^{-\delta\zeta}\right)\right]. \tag{S13}$$

Summing up the integrals (S9)-(S13) yields the final expression (9) for the Brownian motion.

We consider now the velocity autocorrelation function $C(s) \sim \alpha(\alpha - 1)D_\alpha s^{\alpha-2}$. Assuming δ large enough to ensure $\int_0^\delta ds\, C(s) \to \alpha D_\alpha \delta^{\alpha-1}$, the integrals in (S8) can be solved explicitly:

$$\int_0^\delta ds\, C(s) \left(\Delta - \frac{\delta}{3}\right) \simeq \alpha D_\alpha \delta^{\alpha-1}\left(\Delta - \frac{\delta}{3}\right), \tag{S14}$$

$$\int_\delta^\Delta ds\, C(s)\,(\Delta - s) = D_\alpha\left[\Delta^\alpha - \alpha\Delta\delta^{\alpha-1} + (\alpha - 1)\delta^\alpha\right]; \tag{S15}$$

$$\int_0^\delta ds\, C(s) s^2 \left(\delta - \frac{s}{3}\right) = D_\alpha \frac{\alpha(\alpha-1)(2\alpha+5)}{3(\alpha+1)(\alpha+2)}\delta^{2+\alpha}; \tag{S16}$$

$$\int_{\Delta-\delta}^{\Delta} ds\, \frac{C(s)}{6}\Big(\Delta-\delta-s\Big)^3 = \frac{\alpha(\alpha-1)D_\alpha}{6}\Bigg[-\frac{6(\Delta-\delta)^{\alpha+2}}{(\alpha-1)\alpha(\alpha+1)(2+\alpha)}+ \frac{\Delta^{\alpha-1}}{\alpha-1}(\Delta-\delta)^3-\frac{3\Delta^\alpha}{\alpha}(\Delta-\delta)^2+ \frac{3\Delta^{\alpha+1}}{\alpha+1}(\Delta-\delta)-\frac{\Delta^{\alpha+2}}{\alpha+2}\Bigg]; \tag{S17}$$

$$\int_{\Delta}^{\Delta+\delta} ds\, \frac{C(s)}{6}\Bigg(\Delta+\delta-s\Bigg)^3 = \frac{\alpha(\alpha-1)D_\alpha}{6}\Bigg[\frac{6(\Delta+\delta)^{\alpha+2}}{(\alpha-1)\alpha(\alpha+1)(2+\alpha)}- \frac{\Delta^{\alpha-1}}{\alpha-1}(\Delta+\delta)^3+\frac{3\Delta^\alpha}{\alpha}(\Delta+\delta)^2- \frac{3\Delta^{\alpha+1}}{\alpha+1}(\Delta+\delta)+\frac{\Delta^{\alpha+2}}{\alpha+2}\Bigg]. \tag{S18}$$

Inserting the integrals (S14)-(S18) into Eq.(S8) yields the expression

$$\ln\frac{S(\Delta)}{S(0)} = -\frac{\gamma^2 g^2 D_\alpha}{(\alpha+1)(\alpha+2)}\Bigg[(\Delta+\delta)^{\alpha+2}+ (\Delta-\delta)^{\alpha+2}-2\Delta^{\alpha+2}-2\delta^{\alpha+2}\Bigg], \tag{S19}$$

corresponding to the result reported in Ref.[1], obtained using the position correlation function (see Sec.2).

We now want to estimate the error that one makes assuming the approximated Eq.(10) instead of the correct form (S19). If the NMR attenuation in Eq.(S19) is denoted by I, and that in Eq.(10) by I_{app}, we evaluate the relative error as

$$\Delta S = \frac{|I_{app}-I|}{I}. \tag{S20}$$

The analysis of ΔS for different values of δ and α is shown in Fig.S3, as a function of the delay time Δ. The relative error is $\leq 5\%$ for any standard NMR measurements conditions, strengthening the validity of the approximation (10).

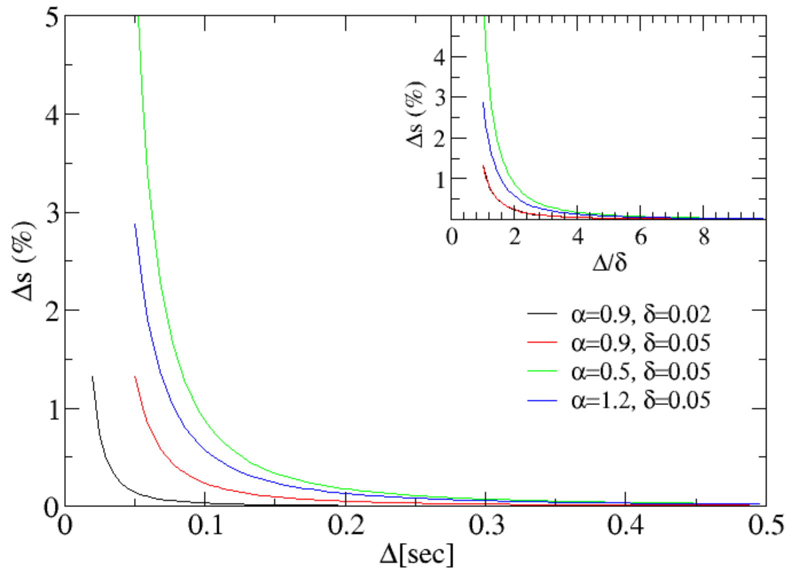

Figure S3: Relative error ΔS (Eq.(S20)). The relative error between the approximated expression I_{app} and the formally correct expression I is shown as a function of Δ for various values of δ an α. Inset: same quantity as in the main panel but plotted versus the rescaled variable Δ/δ.

2 Calculation of NMR signal attenuation in PFG experiments: position autocorrelation function.

The second order cumulant expansion can be performed also by adopting the phase definition $\phi(t) = \gamma \int_0^t dt' \mathbf{r}(t') \cdot \mathbf{G}(t')$ [2, 3, 4, 5, 6, 1, 7, 8]

$$\ln \frac{S(TE)}{S(0)} \simeq -\frac{\gamma^2}{2} \int_0^{TE} dt_1 \int_0^{TE} dt_2 \langle x(t_1) x(t_2) \rangle G(t_1) G(t_2), \tag{S21}$$

where $\langle x(t) \rangle = 0$ (stagnant liquids). Once one assumes the stationarity of the position autocorrelation function, the analogous of Eq.(5) reads

$$\ln \frac{S(TE)}{S(0)} \simeq -\gamma^2 \int_0^{TE} \langle x(0) x(s) \rangle ds \int_s^{TE} G(t') G(t'-s)\, dt'. \tag{S22}$$

We study the quantity

$$\hat{I} = \int_0^{TE} G(t) G(t-s) dt, \tag{S23}$$

for different values of the variable $0 < s < TE$ (see Fig.S4).

- $0 \leq s \leq \delta$

$$\int_{t_1+s}^{t_1+\delta} G(t) G(t-s) dt + \int_{t_1+\Delta+s}^{t_1+\Delta+\delta} G(t) G(t-s) dt; \tag{S24}$$

- $\Delta - \delta \leq s \leq \Delta$

$$\int_{t_1+\Delta}^{t_1+\delta+s} G(t) G(t-s) dt; \tag{S25}$$

- $\Delta \leq s \leq \Delta + \delta$

$$\int_{t_1+s}^{t_1+\Delta+\delta} G(t) G(t-s) dt. \tag{S26}$$

By using the definition (S1) we get

$$\ln \frac{S(\Delta)}{S(0)} = -\gamma^2 g^2 \Bigg\{ 2 \int_0^{\delta} ds\, \langle x(0) x(s) \rangle \Big(\delta - s \Big) + \int_{\Delta-\delta}^{\Delta} ds\, \langle x(0) x(s) \rangle \Big(\Delta - \delta - s \Big) - \int_{\Delta}^{\Delta+\delta} ds\, \langle x(0) x(s) \rangle \Big(\Delta + \delta - s \Big) \Bigg\} \tag{S27}$$

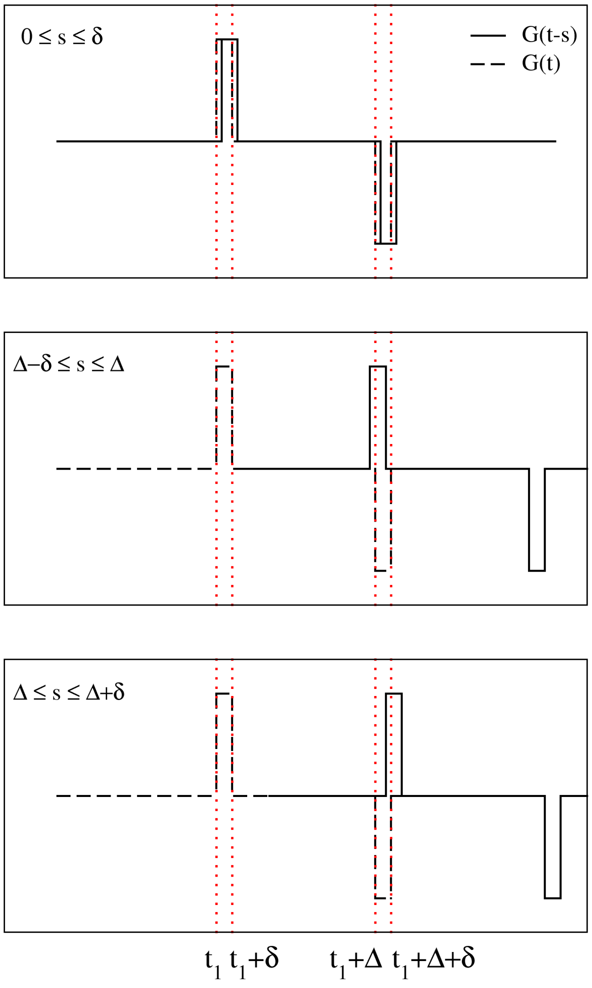

Figure S4: Graphical representation of the contributions in the Eq.(S23). The quantities $G(t)$ and $G(t-s)$ are shown for different values of s. The three contributions to the terms expressed mathematically in Eqs.(S24)-(S26).

We now proceed to the derivation of the anomalous formula Eq.(S19) from the Eq.(S27). First of all we notice that the position autocorrelation function of a system exhibiting the mean square displacement $\langle [x(\Delta) - x(0)]^2 \rangle = 2D_\alpha t^\alpha$ and the velocity autocorrelation function $\alpha(\alpha-1)D_\alpha s^{\alpha-2}$ is

$$\langle x(0)x(s)\rangle = -D_\alpha s^\alpha. \tag{S28}$$

Then, the former expression yields

$$2\int_0^\delta ds\, \langle x(0)x(s)\rangle\, (\delta - s) = D_\alpha \frac{-2\delta^{2+\alpha}}{(1+\alpha)(2+\alpha)}; \tag{S29}$$

$$\int_{\Delta-\delta}^{\Delta} ds\, \langle x(0)x(s)\rangle \Big(\Delta - \delta - s\Big) = D_\alpha \left[\frac{(\Delta-\delta)^{2+\alpha} - \Delta^{2+\alpha}}{(1+\alpha)(2+\alpha)} + \frac{\delta\Delta^{1+\alpha}}{1+\alpha}\right] \tag{S30}$$

$$-\int_{\Delta}^{\Delta+\delta} ds\, \langle x(0)x(s)\rangle \Big(\Delta + \delta - s\Big) = D_\alpha \left[\frac{(\Delta+\delta)^{2+\alpha} - \Delta^{2+\alpha}}{(1+\alpha)(2+\alpha)} - \frac{\delta\Delta^{1+\alpha}}{1+\alpha}\right] \tag{S31}$$

Summing the previous contributions as in Eq.(S27) yields the Eq.(S19).

3 Variance gamma Process

We simulated the Variance Gamma Process (VGP) [9] without drift, a stochastic process with independent increments distributed according to the variance-gamma distribution:

$$v(t) = \xi_{VGP}(t), \tag{S32}$$

with $\langle \xi_{VGP} \rangle = 0$ and $\langle \xi_{VGP}(t)\xi_{VGP}(t')\rangle = \sigma^2 \Delta\tilde{G}\delta(t-t')$ where σ is a constant and $\Delta\tilde{G} \sim \Gamma(\Delta t, 1)$ is an independent Gamma distributed increment [10]. Hence, albeit stationary, the VGP clearly does not fulfill the hypothesis (7) . Thus the process exhibits normal diffusion

$$\langle [x(t) - x(0)]^2 \rangle = \sigma^2 t \tag{S33}$$

but it is not Gaussian [9], i.e.

$$\langle [x(t) - x(0)]^4 \rangle = 3\sigma^4 t(\nu + t), \tag{S34}$$

We have simulated five synthetic DW-NMR signals for the values of δ, g and $D = \sigma^2/2$ reported in Table 3 and displayed in Fig.S5. We used the formula (22) to fit the numerical curves. At first, we notice that the fitting curves (dashed lines) reproduce remarkably well the synthetic ones. However, a close inspection of the values of the parameters D and ζ obtained by fit, reveals that they differ considerably from those expected. As a matter of fact, in Table 3 it is shown that the the fitted D is underestimated of nearly one order of magnitude compared to the true value implemented, for any δ. The value of ζ fulfills the condition $\delta\zeta \gg 1$ which would ensure that the velocity correlation function is a delta function.
The VGP trajectories have been obtained as Brownian motion without drift and subjected to a random time changes which follow a gamma process. The simulation protocols requires that the time interval $[0, 1]$ is subdivided into N independent increments of amplitude $\Delta t = 10^{-3}$ *sec*, i.e. $0 = t_0 < t_1 < ... < t_{N-1} < t_N = 1$. Hence the position is implemented according to the following equation

$$x(t_i) = x(t_{i-1}) + \sigma\sqrt{\Delta\tilde{G}_i}\tilde{\zeta}_i \tag{S35}$$

where $\tilde{\zeta}_i$ is a Gaussian noise satisfying $\langle\tilde{\zeta}_i\rangle = 0$ and $\langle\tilde{\zeta}_i\tilde{\zeta}_j\rangle = \delta_{ij}$. The number of trajectories in the statistical ensemble is $N_t = 2 \cdot 10^4$.

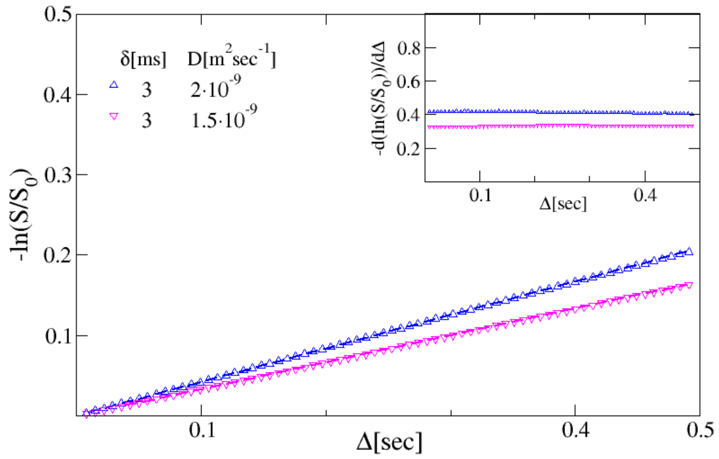

Figure S5: VGP echo amplitudes. Main panel: comparison between different synthetic NMR signals obtained from VGP trajectories (symbols) and fitting curves (dashed curves). The signals are obtained with a gradient $g = 0.02\ T/m$. The fitted values of the VGP constants (D and ζ)are reported in Table 3. Inset: trend of the derivative of the logarithm of the normalized NMR signals shown in the main plot. The outcome is constant as for any diffusive process.

4 Supplementary figures

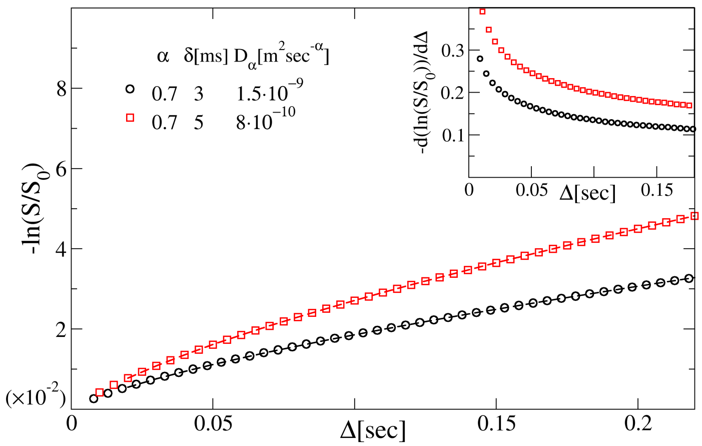

Figure S6: Anomalous diffusion echo amplitudes. Main plots: comparison between different synthetic NMR signals obtained from FBM subdiffusive trajectories (symbols), and relative fitting curves (dashed curves). The signals are obtained with a gradient $g = 0.01\ T/m$. The fitted values of the FBM constants are reported in Table 4. Inset: trend of the derivative of the logarithm of the normalized NMR signals shown in the main plot. Superdiffusive (increasing) and subdiffusive (decreasing) systems are easily appraised.

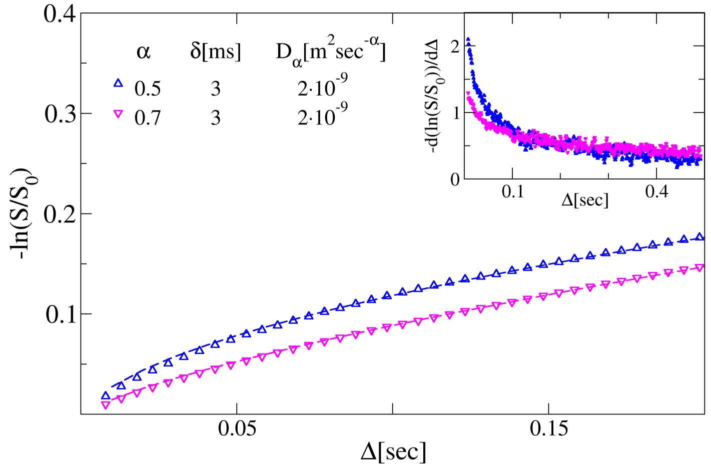

Figure S7: CTRW echo amplitudes. Main plot: comparison between different synthetic NMR signals obtained from CTRW trajectories (symbols) and fitting curves (dashed curves). The signals are obtained with a gradient $g = 0.02\ T/m$. The fitted values of the CTRW constants (D_α and α) are reported in Table 5. Inset: trend of the derivative of the logarithm of the normalized NMR signals shown in the main plot.

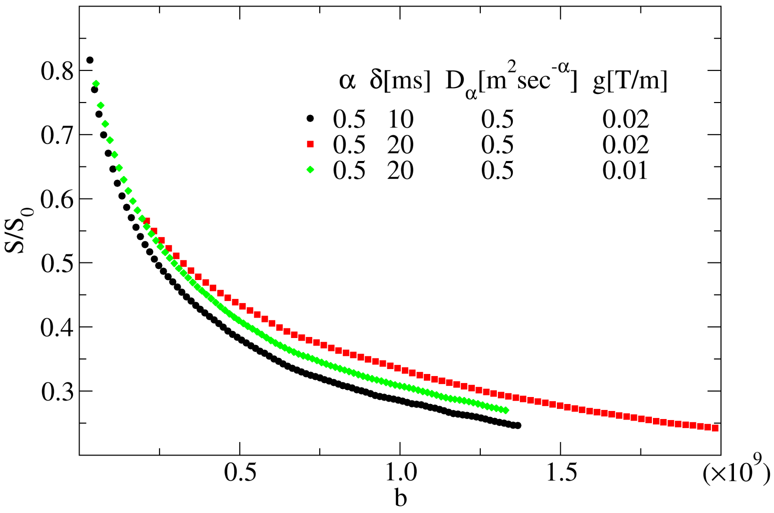

Figure S8: Behavior of the synthetic rescaled DW-NMR signals as a function of the parameter $b = (\gamma g \delta)^2(\Delta - \delta/3)$ for CTRW synthetic signals. The corresponding parameters are displayed in the legend. The plot shows non-collapsing curves.

5 Rician noise effect

To show the noise effect to the practicability of the validation rules, we added some Rician noise to the FBM and CTRW synthetic signals and we performed the rescaling of Fig. 4. The noisy signal was obtained from the original signal S by the relation

$$\sqrt{\left(S + \frac{\eta}{SNR}\right)^2 + \left(\frac{\eta}{SNR}\right)^2} \qquad \text{(S36)}$$

where η is a white noise with zero mean and variance equal to 1, and SNR is the signal-to-noise ratio.

To obtain the final signal we have averaged on 50 realizations of noise.

The results are displayed in Fig.S9 and show collapsing curves for FBM synthetic signals for times below Δ^*, while the CTRW synthetic signals remain significantly separate.

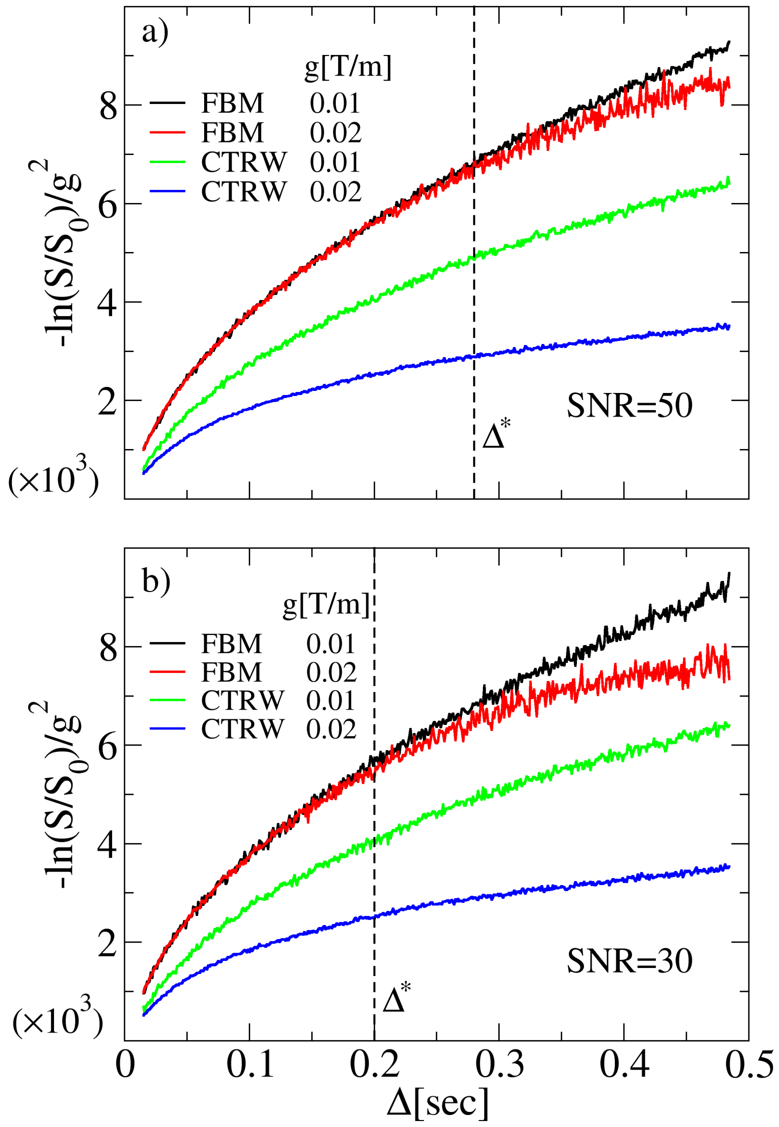

Figure S9: Behavior of the synthetic rescaled DW-NMR signals for FBM and CTRW cases in presence of Rician noise [11]. The parameters used are $\alpha = 0.5$, $D_\alpha = 2 \cdot 10^{-9} m^2\ s^{-\alpha}$ and $\delta = 0.01\ s$ while the gradient intensity is displayed in legend for the various cases. The SNR parameter is equal to 50 in the panel a) and 30 in the panel b). The plots show collapsing curves for FBM synthetic signals for times below Δ^*.

References

[1] J. Kärger, H. Pfeifer, and G. Vojta, "Time correlation during anomalous diffusion in fractal systems and signal attenuation in nmr field-gradient spectroscopy," *Physical Review A*, vol. 37, no. 11, p. 4514, 1988.

[2] J. Stepišnik, "Analysis of nmr self-diffusion measurements by a density matrix calculation," *Physica B+ C*, vol. 104, no. 3, pp. 350–364, 1981.

[3] J. Stepišnik, "Measuring and imaging of flow by nmr," *Progress in Nuclear Magnetic Resonance Spectroscopy*, vol. 17, pp. 187–209, 1985.

[4] D. Sheltraw and V. Kenkre, "The memory-function technique for the calculation of pulsed-gradient nmr signals in confined geometries," *Journal of Magnetic Resonance, Series A*, vol. 122, no. 2, pp. 126–136, 1996.

[5] L. Wang, A. Caprihan, and E. Fukushima, "The narrow-pulse criterion for pulsed-gradient spin-echo diffusion measurements," *Journal of Magnetic Resonance, Series A*, vol. 117, no. 2, pp. 209–219, 1995.

[6] P. T. Callaghan, *Principles of nuclear magnetic resonance microscopy.* Oxford University Press on Demand, 1993.

[7] V. Lisỳ and J. Tóthová, "Nmr signals within the generalized langevin model for fractional brownian motion," *Physica A: Statistical Mechanics and its Applications*, vol. 494, pp. 200–208, 2018.

[8] V. Lisỳ and J. Tóthová, "Attenuation of the nmr signal in a field gradient due to stochastic dynamics with memory," *Journal of Magnetic Resonance*, vol. 276, pp. 1–6, 2017.

[9] D. B. Madan, P. P. Carr, and E. C. Chang, "The variance gamma process and option pricing," *Review of Finance*, vol. 2, no. 1, pp. 79–105, 1998.

[10] M. C. Fu, "Variance-gamma and monte carlo," in *Advances in Mathematical Finance*, pp. 21–34, Springer, 2007.

[11] R. M. Henkelman, "Measurement of signal intensities in the presence of noise in mr images," *Medical physics*, vol. 12, no. 2, pp. 232–233, 1985.
